# Synthesis and characterization of NIR-sensitive curcumin-gelatin nanoparticles for targeted drug delivery in 3D colon cancer

**DOI:** 10.1038/s41598-026-42199-3

**Published:** 2026-03-05

**Authors:** Dilşad Özerkan, Ferdane Danışman-Kalındemirtaş, İshak Afşin Kariper

**Affiliations:** 1 Faculty of Engineering and Architecture, Department of Genetic and Bioengineering, Kastamonu University, Kastamonu, Turkey; 2 Faculty of Medicine, Department of Physiology, Erzincan Binali Yıldırım University, Erzincan, Turkey; 3 Faculty of Medicine, Department of Physiology, Bilecik Şeyh Edebali University, Bilecik, Turkey; 4 Education Faculty, Department of Science Education, Erciyes University, Kayseri, Turkey

**Keywords:** Curcumin, Gelatin nanoparticles, Infrared light (IR), Colon cancer 3D model, Research and development (R&D), Biochemistry, Biotechnology, Cancer, Chemistry, Drug discovery, Nanoscience and technology

## Abstract

Curcumin (CUR) exhibits potent anti-cancer effects; however, its poor water solubility limits its clinical use. This study explores gelatin nanoparticles (GelNPs) as nanocarriers to improve curcumin delivery. We aimed to enhance the release and efficacy of photosensitive curcumin-loaded GelNPs (Cur-GelNPs) using infrared (IR) light-induced localized hyperthermia in a 3D co-culture cancer model. Cur-GelNPs were synthesized and characterized via DLS, FTIR, STEM, Raman, HPLC, and DSC. Cytotoxicity, migration, invasion, apoptosis, and drug resistance were assessed using MTT, Transwell, Hoechst/PI staining, and Rho123 assays, respectively. Optimal results were achieved by IR treatment at 38 °C for 30 s. Cur-GelNPs localized in the cytoplasm due to curcumin’s natural fluorescence. At 25 µg/ml, Cur-GelNPs significantly reduced the viability, invasion, and migration of colon cancer cells, while promoting apoptosis and mitochondrial damage—more effectively when combined with IR. Importantly, minimal toxicity was observed in healthy cells, suggesting selective cancer targeting. This method enables spontaneous drug-macromolecule binding, reducing time, cost, and labor. Overall, IR-activated Cur-GelNPs represent a promising approach for targeted colon cancer therapy and may offer broader contributions to nanomedicine and drug delivery systems.

**Supplementary Information:**

The online version contains supplementary material available at 10.1038/s41598-026-42199-3.

## Introduction

Colorectal cancer (CRC) is one of the most common cancers worldwide and a significant cause of mortality in both developed and developing countries. According to the World Health Organization (WHO), about 1.9 million new cases of colon cancer are diagnosed each year and more than 900,000 people die from this disease^1^. Conventional treatment methods include surgical resection, chemotherapy and radiotherapy. However, these methods often encounter severe limitations such as systemic toxicity, drug resistance and relapse after treatment^2^. For these reasons, efforts are being made to develop next-generation therapeutic strategies that are more targeted, highly effective and have fewer side effects in the treatment of colon cancer.

In recent years, nanotechnology-based drug delivery systems and intelligent therapeutic platforms have shown great potential in this field. Micro- and nanoscale delivery platforms (MNDP), as one of the pioneers of this innovative approach, offer advantages such as tumor-specific control of the microenvironment, precise targeting and controlled drug release^3^. These structures, which can be controlled by external stimuli such as magnetic fields, enable the focused treatment of cancerous tissue using a minimally invasive approach. In 2014, Gao and Wang gave a brief overview of advances in in vitro active drug delivery, focusing mainly on MNDP activated by chemical reactions and external fields^4^. With the recent development of MNDP active drug delivery has shifted from the test tube to the cellular level and living animal models. The first example of the use of chemically triggered microrobots for active drug delivery in living mice was identified by Gao et al. 2015^5^. In 2016, it was then shown that magneto-aerotactic bacteria functionalized with nanoliposomes can be efficiently transported into hypoxic tumor areas using external magnetic fields^6^. To increase their efficacy, MNDP need to be integrated with biocompatible and valuable materials. The studies conducted have shown that the ability of drug delivery systems to achieve controlled release at the targeted site while exerting an appropriate level of cytotoxicity constitutes a critical strategy for therapeutic success. Gelatin-based nanostructures are among the biopolymer platforms that are currently gaining interest. Gelatin is considered a safe and effective drug carrier due to its easy biodegradability in the human body^7^. Gelatin is produced by hydrolyzing collagen, which denatures the collagen and causes it to lose its triple helix structure. Collagen is hydrophobic and is therefore hydrolyzed to gelatin under both alkaline and acidic conditions. During gelation, a conformational rearrangement of the gelatin chains occurs, partially restoring the triple helix structure. As a result, two types of gelatin are formed: type A by acid hydrolysis and type B by base hydrolysis. Gelatin has two different isoelectric points: Type A and Type B. The isoelectric point of type A is in the range of 7–9, while that of type B is in the range of 4–5. As a result, nano-sized gelatins can yield a variety of nanoparticles^8^.

On the other hand, hyperthermia can facilitate controlled drug release by inducing temperature-dependent structural changes in drug carrier systems (such as thermosensitive gelatin nanoparticles), thereby enabling faster or more controlled release of the drug at the target site. This approach enhances targeting accuracy while reducing systemic side effects. It can be used as an adjunct to established oncology treatments, such as chemotherapy and radiotherapy, and improves drug delivery by increasing cellular permeability in the tumor microenvironment^9,10^. Hyperthermia enhances oxygenation by increasing blood flow to tumor tissue, thereby potentially increasing the efficacy of radiation^11^. Additionally, heat facilitates the penetration of various chemotherapeutic agents into cells and enhances their ability to cause DNA damage^12^. In recent years, nanotechnology-assisted hyperthermia applications have gained in importance. Targeted hyperthermia, for example, utilizes photothermal agents (such as gold nanoparticles, graphene oxide, and curcumin derivatives) to heat only the tumor area without damaging surrounding healthy tissue^13^. These techniques enable a more targeted and regulated treatment^14^.

Infrared light, especially near-infrared (NIR) light (650–950 nm), is often used for photothermal therapy due to its ability to penetrate deeply into biological tissue. Photothermal agents, which generate heat when triggered by NIR light, are directed to the tumor site and cause local hyperthermia, leading to apoptosis or necrosis of cancer cells^15^. The NIR-II range (1000–1700 nm) has recently attracted significant attention due to its lower scattering and enhanced tissue penetration. Due to its minimally invasive nature and tailored effect, NIR-guided hyperthermia has shown promising results in numerous preclinical studies^16^. In combination therapy, NIR light-induced hyperthermia can cause drug-carrying nanoparticles to release their contents. This demonstrates the synergistic effect of photothermal therapy and drug release, which improves therapeutic efficacy^17^. For example, in studies using curcumin-containing nanoparticles triggered with NIR light to achieve both photothermal and chemotherapeutic effects, cell death rates in colorectal cancer cells were significantly increased^18^. Curcumin is a natural polyphenol with well-documented anti-cancer properties that make it an essential therapeutic candidate against colorectal cancer cells, as it suppresses cell proliferation, inhibits metastasis and induces apoptosis, among other effects^19^. However, bioavailability issues limit the successful therapeutic use of curcumin. This hurdle can be overcome by administering curcumin in the form of gelatin-based nanoparticles. The temperature regulation of gelatin molecules enables nanoscale production^20^. Moreover, when curcumin interacts with gelatin, it surrounds the gelatin molecule and binds easily to its hydrophobic corners^21^.

This study focuses on the targeted and controlled delivery of curcumin-loaded gelatin-based nanoparticles to 2-dimensional (2D) colorectal cancer cells and 3-dimensional (3D) colon cancer/endothelial co-culture models using nanodrug carrier systems, aiming to develop a multicomponent therapeutic platform. Several unique aspects distinguish this work from other gelatin–curcumin nanoformulations in the literature^22–24^. The synthesis approach offers a novel production strategy requiring minimum energy, optimizing the sonication time (30 s) and the carrier/drug ratio (1:1). Controlled and accelerated release behavior was achieved at 38 °C by using IR light for temperature-dependent activation of nanoparticles; this IR-triggered release mechanism was demonstrated for the first time in gelatin-based curcumin systems. Nanoparticle behavior was systematically analyzed using a triple characterization approach based on DLS–STEM–HPLC data. The effects of NIR-activated Cur-GelNPs on invasion, migration, and proliferation in a 3D colon cancer–endothelium co-culture model were evaluated for the first time, demonstrating a more physiological model compared to conventional 2D cultures. In light of this information, the rationale for developing Curcumin-Loaded Gelatin Nanoparticles as a controlled drug delivery carrier in our study is detailed below.

## Materials and methods

This study was planned in four main phases: the generation of NIR-responsive curcumin-loaded gelatin nanoparticles as a controlled drug-delivery carrier, their characterization, IR activation, and testing of their biological activity. The methods are presented in the logical order of these phases, with each experimental step laid out as a foundation for the next.

### Synthesis of curcumin-loaded gelatin nanoparticles (Cur-GelNPs) as nanodrug carriers

1 mg/mL gelatin (GEL) (Merck, Germany) was prepared as a stock solution in distilled water. A 1 mg/mL stock solution of curcumin (CUR) (Santa Cruz, USA) was prepared in absolute ethanol. 1 mL of the 1 mg/mL gelatin stock solution and 1 mL of the 1 mg/mL curcumin stock solution were combined in the same medium. For HPLC analysis, the solutions were sonicated in an ultrasonic bath at room temperature for 1, 5, 10, 30, 60, 120, and 180 s. To prepare the carrier/curcumin mixtures, 1 mg/mL of the carrier gelatin stock solution was added to the curcumin stock solution at concentrations of 10%, 25%, 50%, 75%, 100%, 125%, and 150% of the carrier, and the mixtures were stirred. The resulting mixtures were sonicated in an ultrasonic bath for 30 s, which was determined to be the optimum parameter.

For sonication and a 1:1 carrier-to-drug concentration ratio, which are optimal conditions, 1 mL of a 1 mg/mL gelatin stock solution and 1 mL of a 1 mg/mL curcumin stock solution were withdrawn and sonicated in the same medium for 30 s. The mixtures were activated with an near infrared light source at temperatures ranging from 37 °C to 42 °C. A 150W infrared light source (Rotlichtlampe IR 150) was directed at the surface of the samples at an angle of 45° from a distance of 15 cm.

### Characterization and stability evaluation of Cur-GelNPs

DLS and zeta potential values determine the stability of the prepared Cur-GelNPs. Particle size, surface charge and FTIR analyses are used to understand how long they remain stable at different temperatures. A change in zeta charge, an increase in particle size, or the appearance of specific vibrational signals in the FTIR of curcumin would mean that the stability of Cur-GelNP is compromised.

#### DLS analysis

The particle size measurements were performed using a Zetasizer Nano ZS at a wavelength of 633 nm, with a four mW He-Ne laser and a detection angle of 173°, at a temperature of 22–23 °C.

#### FTIR analysis

The infrared spectra of the samples were recorded using a PerkinElmer Spectrum 400 spectrometer with a resolution of 4 cm-1 and a DTGS detector, with 10 scans per spectrum.

#### STEM analysis

GelNPs and Cur-GelNPs samples were dropped onto a glass substrate and coated with 450 nm thick Au/Pd using a small Polaron sc 7620 sputter coater, and then analyzed by STEM. To view liquid samples for analysis, the samples were deposited on glass substrates, dried and then placed in the instrument.

#### Absorption measurements

A Hach Lange DR 5000 spectrophotometer was used to evaluate the absorption of aqueous solutions of plastics. Water was used as a reference for the measurements in the wavelength range of 200–1100 nm.

#### Raman analysis

A WITec Alpha 300 M+ Raman spectrometer operating at 532 nm was used for the Raman measurements. Liquid samples were placed on a glass slide and focused on the sample using a 50X objective (numerical aperture: 0.85; spot size: 2 μm). The surface of each sample was scanned for 10 s (exposure time) using a 3.2 mW laser beam for each spectrum.

#### HPLC analysis

The Agilent Technologies 1260 Infinity HPLC instrument was used to measure the amount of curcumin that was not retained by the carrier in the solution. The amount of curcumin retained by the carrier gelatin was calculated by subtracting the amount of curcumin added from the amount of curcumin measured on the HPLC instrument (i.e., the amount of curcumin released in the solution). The samples were separated on a Supelco Discovery 5 μm C18 (4.6 mm × 5.0 μm) column. The mobile phase was ACN:20 mM (40:60 v/v). The flow rate was 1.5 mL min^–1^. The injection volume was 20 µL and UV detection was performed as follows: Tracking at 262 nm. The peak area was eluted. The amount of curcumin released in the solution was calculated by comparing the areas of the peaks with those of standard curcumin samples.

#### DSC analysis

The liquid samples were analyzed using a Hitachi DSC-7020 instrument. They were heated in aluminum pans at a rate of 1 °C/min in the range of (– 50–300) °C. An indium standard was used to calibrate the instrument and an inert environment with nitrogen gas was provided.

### Studies on curcumin release

Since the amount of curcumin released into the membrane is small, it remains below the HPLC limit of quantification. Therefore, the release values were calculated by absorption measurements in the FTIR spectrum. According to the Lambert-Beer law, the ratio of the infrared absorption intensity of the sample (I/Io) is equal to the molar absorptivity of the sample (ε) multiplied by the concentration of the sample (C) and the path length (L). This equation can also be applied to infrared spectroscopy. In our study, we first investigated the release profile of curcumin from gelatin-based nanocarriers using FTIR spectroscopy with a dialysis membrane in physiological pH (7.4) and acidic pH (5.5) buffer solutions. For this purpose, 1 mg/mL of the solution containing curcumin-loaded nanocarriers was added to the dialysis membrane, and both ends were sealed. The dialysis membrane containing the curcumin-loaded nanoparticles was then placed in buffer solutions at different temperatures. In the study, the release of curcumin was compared at 38 and 40 °C. At this stage, samples were collected at 0, 1, 2, 4, 8, 16, 24, 48, and 72 h, and FTIR measurements were performed. The cumulative curcumin release was calculated according to the following formula^25^.

$$\text{Drug loading efficiency}\:(\%)=100\times (W_{Total\: Drug}-W_{Free\: Drug})/W_{Total\: Drug}.$$

                              W_Total Drug_: the total amount of drug and W_Free Drug_: the free drug.

### Cell culture

HCT116 (Human Colon Ca) (RRID: CVCL_0291), HT-29 (Human Colorectal Carcinoma) (RRID: CVCL_0320) and HUVEC (Human Umbilical Vein Endothelium) (RRID: CVCL_2959) cells were obtained from ATCC. A Hoechst immunostaining for mycoplasma contamination is routinely performed in our cell culture laboratory^26^. Culture medium consisting of Dulbecco’s Modified Eagle Medium high-glucose (DMEM) (Gibco, U.S.A.) supplemented with 10% fetal bovine serum (FBS) (PAN Biotech, USA) and 1% penicillin-streptomycin (Gibco, USA) was used to maintain the cells. Cells were grown at 37 °C in an incubator with 5% CO2 to study the effects of the microenvironment. When the cells in the flasks had reached a sufficient number for experimental use, proliferation, cell death, and migration assays were performed.

#### Determination of cell viability using the MTT test

For the MTT method (Invitrogen, U.S.A.), 10 × 10^3^ cells/well were first seeded in 96-well cell culture dishes and allowed to adhere overnight. To select the appropriate dose, cells were treated with curcumin-loaded gelatin nanoparticles (Cur-GelNPs) at varying concentrations (50, 25, and 12.5 µg/mL) for 24, 48, and 72 h. An NIR beam was used to stimulate the nanocarrier at a distance of 15 cm and an angle of 45° above the seeded wells. Only cells seeded in medium were used as a negative control for cell death (maximum viability). Empty gelatin nanoparticles (50 µg/mL) in curcumin doses (50, 25, 12.5 µg/mL) were used as a negative control. A series of experiments without NIR treatment was also performed for the control, experimental and negative control groups. 100 µL of the medium was added to the wells reserved for the blank experiment. The cells were then incubated at 370 °C, 5% CO_2_ for 24, 48, and 72 h. The MTT chemical was prepared as a stock solution at 5 mg/mL in phosphate-buffered saline (PBS) (Merck, Germany), pH 7.2, filtered, and sterilized. At 24, 48, and 72 h, 10 µL of MTT dye was added to each well, and the cells were incubated at 37 °C for 4 h. To dissolve the formazan crystals, 100 µl of DMSO solution was added to all wells, and the color intensity of the cells was measured at 570 nm in a spectrophotometer. Cell viability was determined by absorbance^27^. The viability of untreated control cells was assumed to be 100% and the viability of cells treated with the drug was calculated using the following formula. Each concentration was repeated in three independent wells within the experiment.

$$\% \text{ Viability}= [100\times (\text{Compound treated cell absorbance mean-blinded mean})/(\text{Control cell absorbance mean-blinded mean})].$$

The dose that kills 50% of the cells (IC_50_) was determined using GraphPad Prism 5.

#### Proliferation, wound healing, migration/invasion in the 3D colorectal cancer co-culture model

For direct co-culture, colon cancer cells (HCT116) and endothelial cells (HUVEC) were cultured in DMEM medium supplemented with 10% FBS at a ratio of 4:1, 1:1, and 1:4. Cell proliferation was assessed after 24, 48 and 72 h using an MTT assay performed separately for all cells^28^. All subsequent analyses were performed in a 4:1 co-culture model and compared using the IC_50_ value. The comparison was performed using Cur-GelNPs without NIR treatment as a negative control.

The wound-healing assay is a standard in vitro technique for studying cell migration in two-dimensional media. In this assay, mechanical damage is first used to create a space between cells distributed in a single layer on the well surface. Since there is only one space into which the cells can move, they start moving towards it. In this way, comparative analyses of the cells’ ability to migrate in two dimensions can be performed^29^. For wound-healing analysis, cells were co-cultured at a 4:1 ratio and seeded into 24-well plates. The experiment was initiated once the cells reached 80% confluence. Cells were treated with curcumin-loaded nanocarriers at the determined IC_50_ dose by IR irradiation for 30 s. After 24 h of incubation, a single wound per well was scored in monolayers using a sterile 200 µL pipette tip. The wells were washed with PBS to remove detached cells. At 0, 24, and 48 h, the wound-healing rate was assessed, and images were captured using a light microscope. The experiment was terminated after 48 h, as the control wells closed at that time. The width of the wound closure was quantified using ImageJ, and diagrams were created.

The Transwell cell migration and invasion assay provides a detailed analysis of the ability of cells to sense a specific chemoattractant and overcome a physical barrier^30^. The Transwell experiments were performed using 8.0 μm polycarbonate cell culture inserts (Transwell Permeable Supports, Sarsted, Germany). The cells were serum-deprived for 24 h before the start of the experiments. For invasion experiments, Geltrex LDEV-free Reduced Growth Factor Basement Membrane Matrix (Thermo Scientific, USA) was also added to the Transwells and incubated for 1 h at 37 °C in the incubator to allow gelation. Colon cancer cells were seeded into the upper chamber of 24-well plates at a density of 4 × 10^4^ cells per well. In the lower wells, 1 × 10^4^ HUVECs were seeded (4:1 co-culture model). The cells were treated with Cur-GelNPs at the determined IC_50_ dose by IR irradiation for 30 s and allowed to migrate to the bottom of the membrane in the lower chamber for 24 h. The comparison was performed with Cur-GelNPs without IR treatment as a negative control. The cells adhered to the top of the filter were then removed by wiping with a cotton swab. The migrated cells on both the top insert and the bottom were washed once with 1x PBS after the medium was collected. After fixation with 4% formaldehyde (Pierce, Thermo Scientific, USA), they were washed 2 times with 1x PBS, stained with crystal violet, washed 3 times with 1x PBS and photographed under an inverted microscope^28^.

### Immunofluorescence staining

Given the shape of the cell, DNA is evenly distributed and nuclei are typically spherical. However, during apoptosis, DNA condenses, leading to a pycnotic appearance. Since this does not occur during necrosis, nuclear differences can be used to distinguish between apoptotic, healthy, and necrotic cells. Nuclear condensation studies can be performed using DNA-binding dyes, such as Hoechst 33342. Hoechst 33342 and propidium iodide staining, combined with flow cytometric and fluorescence imaging analysis, are often used for simultaneous staining to analyze apoptosis stages and cell cycle distribution^31^. Hoechst33342/Propidium iodide (PI) double staining will be performed to detect cell death. In 96-well flat-bottom plates, HCT116, HT29, and HUVEC cells were seeded separately at a density of 1 × 10^4^. Cells were treated with curcumin-loaded nanocarriers at the determined IC_50_ dose, combined with NIR light for 30 s, and compared with Cur-GelNPs without NIR treatment as a negative control. After 24 h, the Hoechst33342 (Sigma-Aldrich, USA) and Propidium Iodide (Invitrogen, USA) staining protocol was used. Supernatants were collected from the working solution at 1 µg/mL, and 50 µL of each stain was added to the wells, which were incubated in the dark at room temperature. Photographs were taken using a BIO-RAD ZOE immunofluorescence microscope^32^.

Mitochondrial changes are crucial indicators that reveal cellular stress and health status. Mitochondria play a role in the apoptosis process and the irreversible step in the apoptotic process is mitochondrial activation^33^. Therefore, it is essential to observe mitochondrial morphological changes to determine the extent of cellular damage. Rho123 (Sigma Aldrich, USA) immunostaining was performed to demonstrate mitochondrial membrane damage. HCT116, HT29, and HUVEC cells were seeded separately at a density of 1 × 10^4^ cells. They were treated with curcumin-loaded nanocarriers at the determined IC_50_ dose, followed by 30 s of NIR irradiation, and compared with Cur-GelNPs without NIR treatment as a negative control. will be treated for 24 h. After 24 h, all the supernatant from the cells was removed using a micropipette. After washing with 1 x PBS, the cell groups were fixed in 4% formalin solution (in 1 × PBS). After 1 h at room temperature, the cells were washed with 1× PBS. Cell groups were stained with Rho123 at 10ug/mL and incubated in the dark at 37 °C for 30 min. Photographs were taken with the Bio-Rad Zoe immunofluorescence microscope^32^.

## Results

In the findings section, the results of the physicochemical characterization of Curcumin-Loaded Gelatin Nanoparticles as a Controlled Drug Delivery Carrier are presented first, followed by an evaluation of their activation and release behavior using NIR, and finally, the biological effects in 2D and 3D cell models are reported. This sequence aims to clarify the relationship between the structural properties and the biological behavior of curcumin-loaded gelatin nanoparticles as a controlled drug-delivery carrier system.

### Characterization and stability evaluation of Cur-GelNPs

#### Sonication of Cur-GelNPs

**Fig. 1:**
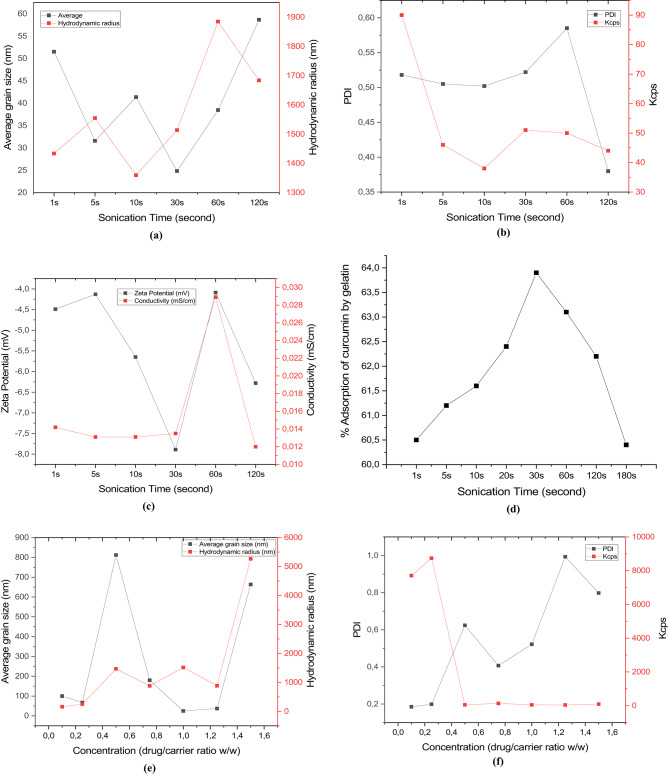

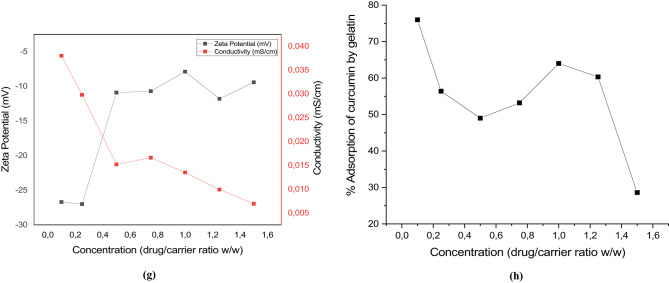
(**a**) Nano-size and hydrodynamic radius of Cur-GelNPs. (**b**) PDI values and Kcps measurements of Cur-GelNPs. (**c**) Zeta potential and conductivity profiles of Cur-GelNPs. (**d**) Percent adsorption of curcumin onto gelatin. (**e**) Nano-size and hydrodynamic radius of Cur-GelNPs in curcumin solution. (**f**) PDI values and Kcps of Cur-GelNPs in curcumin solution. (**g**) Zeta potential, conductivity, and percent adsorption of curcumin by gelatin, reflecting the physicochemical characterization of the nanocarrier system.

The maximum amount of curcumin the nanocarrier can adsorb can be determined from HPLC analysis. At the same time, however, the carrier/curcumin complex must remain nanoscale. The most important parameters for this are the sonication time and the carrier-to-drug concentration ratio. Figure 1a–c shows the results of the DLS measurements, while Fig. 1d shows the percentage of curcumin retained by the gelatin as calculated by HPLC analysis. At sonication times of 1 h, 5 h, 10 h, 30 h, 60 h, and 120 h, the particle size of the complex of the nanocarrier with curcumin was 51-31-41-24-38–58 nm. At the same time, the hydrodynamic radii were 1434, 1555, 1360, 1514, 1885, and 1684 nm, respectively. The PDI values, another critical parameter, were measured to be 0.518, 0.505, 0.505, 0.502, 0.522, 0.585, and 0.380 for sonication times of 1 h, 5 h, 10 h, 30 h, 60 h, and 120 h, respectively. The PDI (Polydispersity Index) is defined as PDI = µ²/Γ and indicates the width of the dispersion (Γ is the average decay rate). According to the device we measured, the particles are volatile when the PDI is greater than 0.725. In other words, although the particle size is in the nanometer range, one cannot speak of a stable nanoparticle. Depending on the sonication time, the PDI values are very close to each other, but are significantly smaller than 0.725. However, the 30-second sonication time with the smallest particle size of 24 nm was selected as the optimal parameter. Below 60 s of sonication, all other sonication times are very close to each other in terms of the hydrodynamic radii. The hydrodynamic radius calculated by the measuring device using the Stokes-Einstein equation reflects the size of the 2nd and, to a lesser extent, the 3rd order. Accordingly, the hydrodynamic radius in the range of 1300–1500 nm was found to be appropriate. At the same time, according to the HPLC results, the highest amount of curcumin in the gelatin nanoparticle was captured after 30 s of sonication, corresponding to a 64% capture rate.

#### Concentration of Cur-GelNPs

Figure 1e–g shows the results of the DLS measurements, while Fig. 1h shows the percentage of curcumin retained by the gelatin as calculated by HPLC analysis. Another important parameter, the concentration ratio of curcumin to carrier, was investigated, as shown in Fig. 3. At a curcumin/carrier ratio of 10%-25%-50%-75%-100%-125%-150%-150%, the average particle sizes were 99-67-67-812-180-24-37-663 and the hydrodynamic diameter values were 157-250-1466-880-1514-883-5267 nm. Depending on the sonication parameters, very strong deviations and concentration changes were observed. However, a curcumin/carrier ratio of 1:1 at 30 s of sonication was chosen as the most suitable parameter. The PDI values for curcumin/carrier ratios of 10%-25%-50%-75%-100%-125%-150% were measured to be 0.185-0.199-0.624-0.407-0.407-0.522-0.993-0.798. According to these measurements, values above 1:1 for the curcumin/carrier ratio do not indicate stable nanointegrity. At the same time, according to the HPLC results, the amount of drug adsorbed by the nanocarrier was measured at ratios of 10%, 25%, 50%, 75%, 100%, 125%, and 150%, corresponding to 76%, 56%, 49%, 53%, 53%, 64%, and 60%, respectively. Although 10% curcumin/carrier ratio seems to be the highest amount of adsorbed curcumin, since this amount would not be sufficient for the effect of curcumin on cancer in the subsequent cancer tests, the next parameter with the highest amount, a 1:1 curcumin/carrier ratio and 30 s sonication parameters, was selected as the optimal parameters. In addition, the zeta potentials at a 1:1 curcumin/carrier ratio and 30-second sonication parameters are also among the lowest values at -7.89 mV. The low conductivity of the solutions is also attributed to the zeta potential. The lower the zeta potential, the lower the solution’s conductivity. This is because the surface charge of the particle is also a measure of the particle’s ability to move in the solution at a certain potential difference. If the surface charge is low, the mobility also decreases. But a high negative surface charge is sufficient. This is because the nanocarrier is designed to release in the Krebs area, driven by the infrared light source rather than by an electric current. The encapsulation efficiency can be calculated using the following formula: (Total added curcumin concentration - amount of curcumin released in the medium)/Total added curcumin concentration × 100. Therefore, the percentage adsorption of curcumin by gelatin also indicates the encapsulation values.

**Fig. 2:**
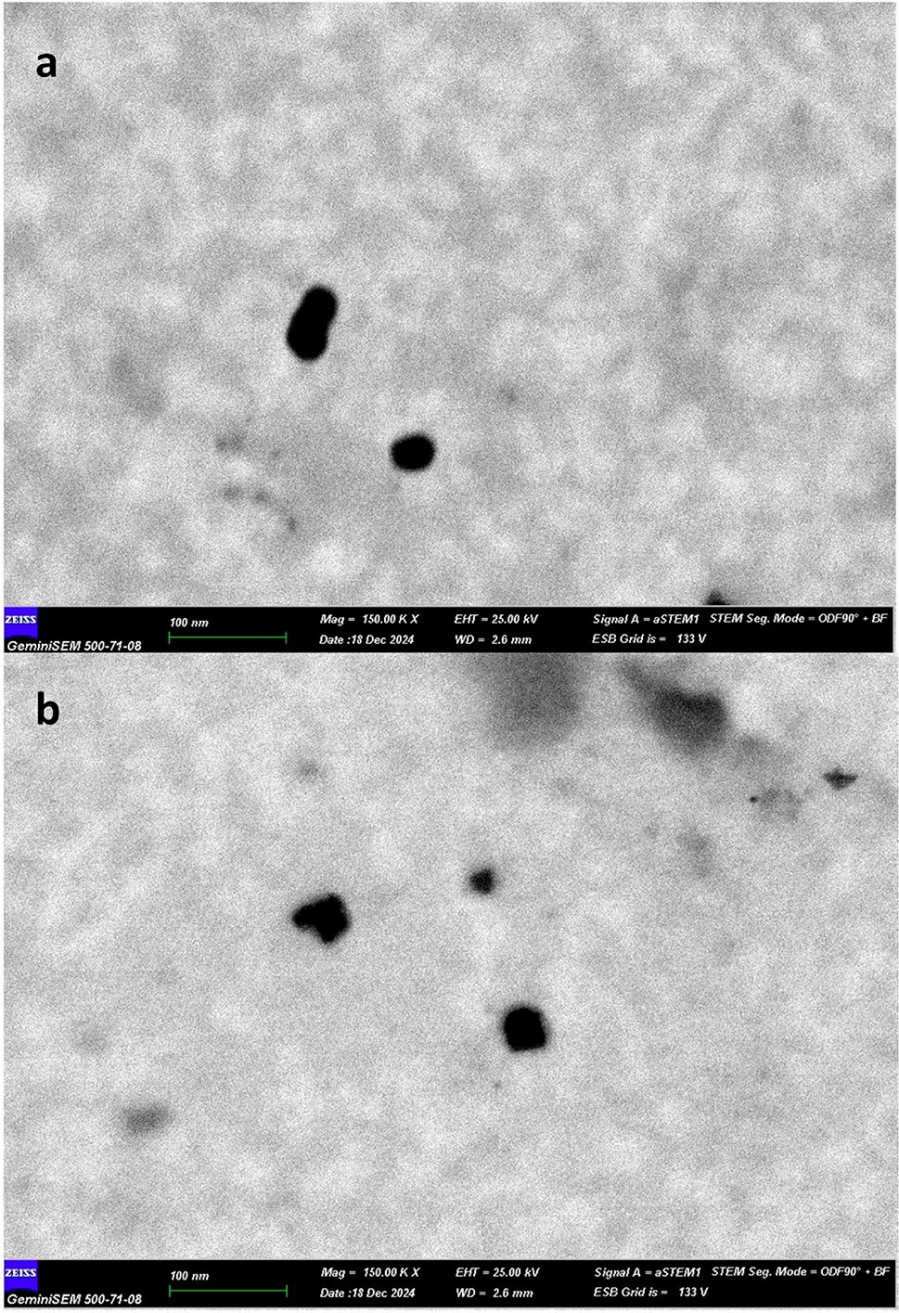
(**a**) STEM image of Cur-GelNPs synthesized under the optimal condition of 30 s sonication, demonstrating uniform nanoparticle morphology. (**b**) STEM image of Cur-GelNPs produced with 30 s sonication and a 1:1 (w/w) curcumin-to-gelatin ratio, showing well-defined nanostructures consistent with optimal formulation parameters.

STEM images were taken to evaluate the morphological expansion and size development observed in the DLS analysis. At 30 s of sonication, which was determined to be the optimum parameter according to the STEM images, one particle of about 20 nm and two particles with a total diameter of 40 nm were detected in Fig. 2a. After the last parameter of 30 s of sonication at a curcumin/carrier ratio of 1:1 (w/w), three different particles with diameters of 10 nm, 20 nm, and 30 nm were observed in Fig. 2b. These results are consistent with the DLS results. The DLS results are in supplementary material 1, and the HPLC results and detailed calculations are in supplementary material 2. After confirming the morphological structure, HPLC was used to determine the curcumin loading capacity of the nanocarriers.

#### Cur-GelNPs activated by an infrared light source

**Fig. 3:**
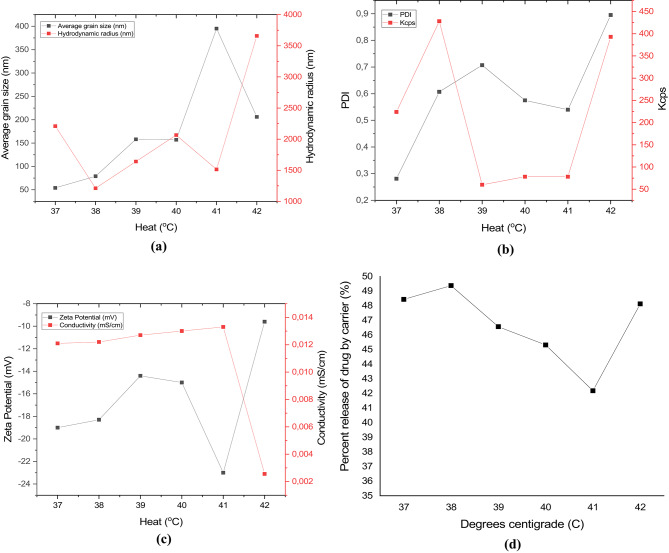
(**a**) Nanoscale dimensions and hydrodynamic radius of the curcumin-loaded nanocarrier. (**b**) PDI values and Kcps measurements of the curcumin nanoparticles, indicating size distribution and stability. (**c**) Zeta potential and conductivity of the nanocarrier dispersed in curcumin solution, reflecting surface charge and colloidal behavior. (**d**) Percentage release profile of curcumin from the nanocarrier system, demonstrating its controlled-release characteristics.

After determining the loading capacity, the temperature-activated release behavior of the nanocarrier was evaluated. Figure 3a–c shows the results of the DLS measurements, while Fig. 1g shows the percentage of active ingredient curcumin released from the carrier gelatin as a function of temperature, calculated by HPLC analysis. While the average particle size at optimal parameters was 24 nm at room temperature (carrier/curcumin ratio 1:1, 30 s sonication, room temperature: 22 °C), the temperature of the Cur-loaded GelNP mixture excited by infrared light increased and was measured to be 54-79-158-157-395–206 nm at 37–38 °C-39–40 °C-41–42 °C. While the hydrodynamic radius at optimal parameters was 1514 nm at room temperature, it was estimated to be 2209-1212-1640-2065-1514–3658 nm at temperatures ranging from 37 to 42 °C. When the carrier curcumin was adsorbed at optimum parameters at room temperature, the PDI value was 0.522. In contrast, at temperatures ranging from 37 to 42 °C, the measured PDI values were 0.281, 0.607, 0.707, 0.707, 0.757, 0.757, 0.540, and 0.895, respectively. This means that the nano stability deteriorates when the nanocarrier swells under the influence of heat. Only the PDI value at 41 °C showed a deviation from the expected value. While the zeta potential was − 7.89 mV when the carrier adsorbed curcumin at optimal parameters at room temperature, it was measured to be (-19)-(-18.3)-(-14.4)-(-15)-(-23)-(-9.6) mV at 37–38 °C-39–40 °C-41–42 °C, respectively. This means that the second layer grows with increasing hydrodynamic radius, leading to a negative increase in surface charge. Particles of 150–200 nm and below are suitable for penetrating the cell membrane. According to the initial parameters, the nanocarrier and curcumin can easily penetrate the cell membrane at a particle size of 24 nm. However, when the nanoparticle is activated with infrared light, it is advisable to activate the nanocarrier at 37 °C and 38 °C to prevent issues during transport. However, according to the HPLC results, the highest release of the nanocarrier-curcumin was approximately 50% at 38 °C. The most suitable release temperature was determined to be 38 °C. In other words, the nanocarrier adsorbs 1 mg/mL of curcumin at 64% releases only 0.64 mg/mL of curcumin, while at 38 °C, it releases only 0.32 mg/mL of it to the environment (Supplementary Material 3).

**Fig. 4:**
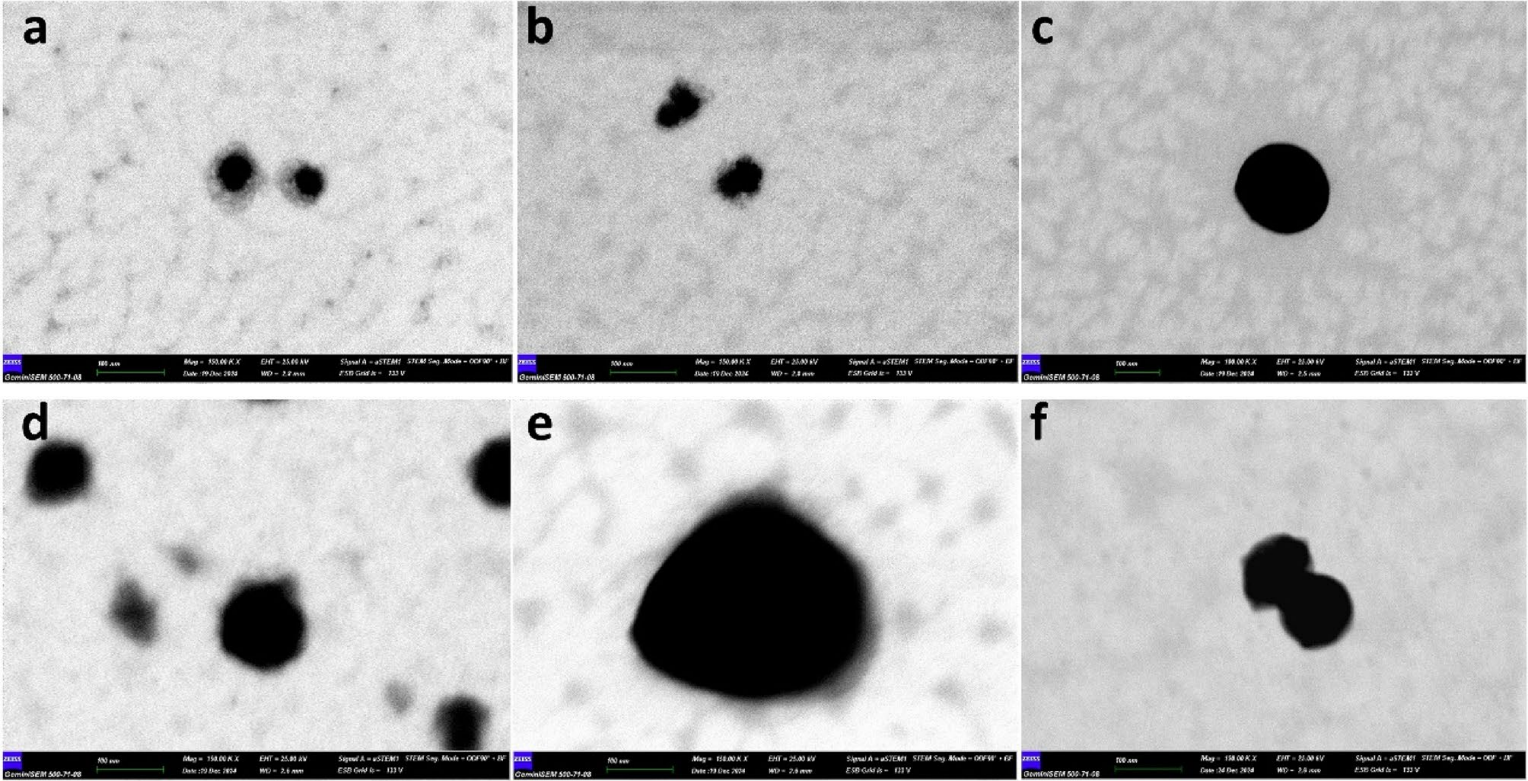
STEM images of Cur-GelNPs captured at varying temperatures: (**a**) 37 °C, (**b**) 38 °C, (**c**) 39 °C, (**d**) 40 °C, (**e**) 41 °C, and (**f**) 42 °C. The images illustrate temperature-dependent changes in nanoparticle morphology and structural integrity.

Figure 4a–f shows the STEM images of the nanocarrier at 37–38 °C-39–40 °C-41–42 °C. According to the DLS results, particle sizes of 54-79-158-157-395–206 nm were measured at temperatures of 37 °C, 38 °C, 39 °C, 40 °C, 41 °C, and 42 °C. These values correspond to approximately 40, 60, 160, 160, 130, 300, and 180 nm, as determined by the STEM images. These results roughly confirm the DLS results. The difference between the DLS results and the STEM results could be due to molecular shrinkage, especially after the solvent is removed from the environment, as well as the molecular shrinkage that occurs after the applied temperature is reached, given that the samples cool down during the long preparation time for STEM.

### The release of curcumin by Cur-GelNPs

**Fig. 5:**
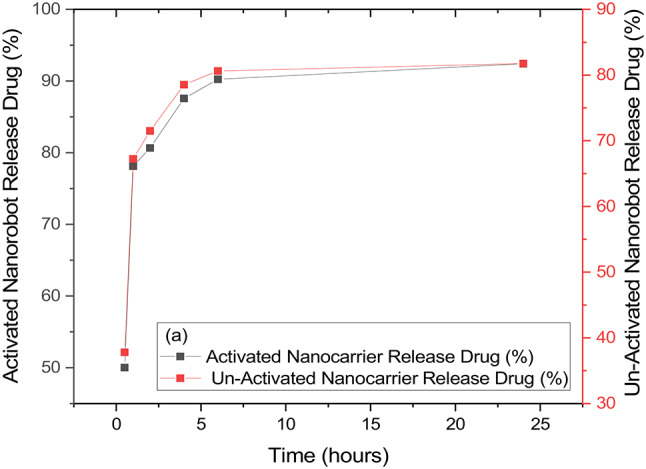
Comparison of curcumin release profiles between non-activated nanocarriers and IR-activated nanocarriers. IR stimulation markedly enhances the release rate, demonstrating the system’s responsiveness to external activation and its potential for controlled, on-demand drug delivery.

Additionally, we investigated whether there is a difference in release when the nanocarrier is activated with near infrared light compared to when it is not activated. Accordingly, the activated nanocarrier released 50-78-81-87-90-93% of the entrapped curcumin after 30 min, 1 h, 2 h, 4 h, 4 h, 6 h, and 24 h, respectively. Without activation, the release of curcumin in 30 min–1 h-2 h–2 h-4 h–4 h-6–24 h was 37-67-71-78-80-82%. To better understand the phenomenon, we compare the percentage release of curcumin from the activated Cur-GelNPs with that from the non-activated Cur-GelNPs, arriving at 1.35, 1.16, 1.14, 1.11, 1.12, and 1.13 in 30 min, 1 h, 2 h, 4 h, 6 h, and 24 h, respectively (Fig. 5). This means that the nanocarrier released by near infrared light was activated very quickly and released 1.35 times more curcumin in the first half hour than the non-activated nanocarrier. As the release continued, this ratio remained constant in the range of 1.11–1.14. The nanocarrier can therefore be easily activated by infrared light and the release of curcumin can be controlled (Supplementary Material File 4).

### Characterization of Cur-GelNPs

**Fig. 6:**
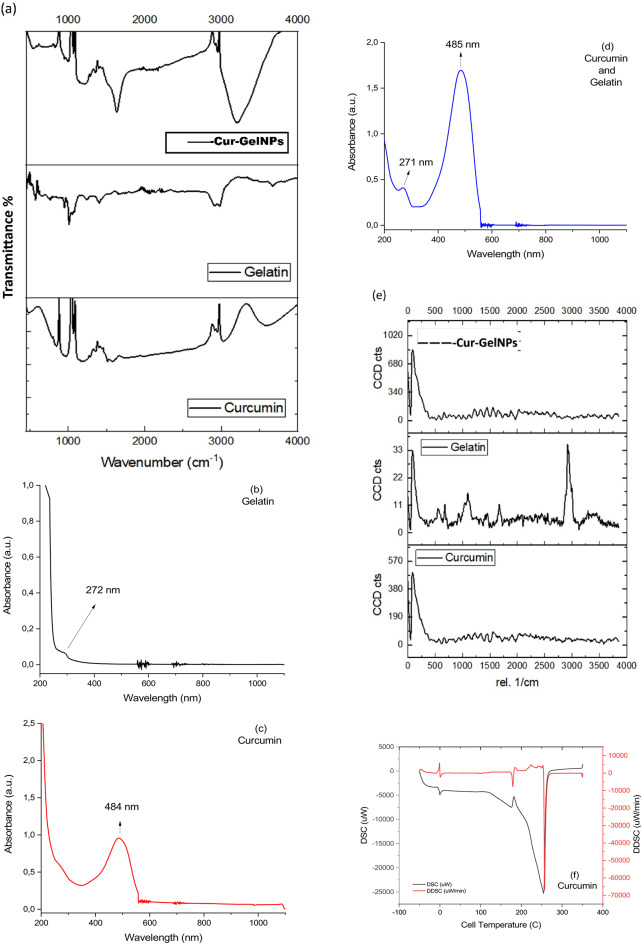

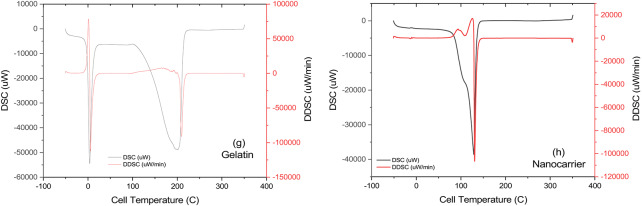
(**a**) FTIR spectrum of Cur-GelNPs, illustrating characteristic functional groups. (**b**–**d**) UV-Vis spectra of gelatin (**b**), curcumin (**c**), and Cur-GelNPs (**d**), showing successful incorporation of curcumin into the nanocarrier. (**e**) Raman spectrum of the Cur-GelNPs, confirming structural features of the nanocarrier. (**f**–**h**) DSC thermograms of curcumin (**f**), gelatin (**g**), and Cur-GelNPs (**h**), indicating thermal behavior and interactions within the nanoparticle system.

Figure 6a shows the FTIR spectra of gelatin, curcumin and the nanocarrier complex formed by both. The NIR spectrum of gelatin indicates that the vibrational signals at 3667 cm^-1^ originate from the amide I and amide II bands, respectively. Amide-I, C = C bond extension; amide-II, N-H vibrational groups and stretching vibrations of the C-N groups of the amide proteins contributed to these bands. Symmetric C-H stretching at ~ 2921–2947 cm^− 1^ and symmetric and asymmetric vibrational bands of -C-H groups at 1399 cm^− 1^ were detected^34,35^. A broad vibrational band at 3575 cm^− 1^ belonging to amine groups was observed in the FTIR spectrum of curcumin. A stretching vibration at 3035 cm^− 1^ attributed to O-H groups and a band with high intensity at 1589 –1521 cm^− 1^ were attributed to mixed vibrations, including the stretching vibrations of the carbonyl bond. The significantly intense band at 1270 cm^− 1^ is also attributed to the bending vibration of the ν(C-O)-phenol band^36^. In the gelatin-curcumin nanocarrier complex, some of these vibrational bands were detected, while other vibrational bands influenced others. However, the most essential band is a powerful vibrational band at 1635 cm^− 1^. This vibrational band also overlapped with the carbonyl vibrational band of curcumin at 1589 –1521 cm^− 1^, resulting in a powerful vibrational band. Considering these data, it is difficult to claim that a chemical bond exists between gelatin and curcumin.

Figure 6b–d shows the UV-VIS spectra of gelatin, curcumin and the nanocarrier complex formed by both. The solution of gelatin in 1 mg/mL distilled water exhibited a maximum absorption band at 272 nm. In fact, gelatin shows two absorption bands at 220 nm due to the peptide bond and the side chains of the aromatic groups (π → π* transition) and at 280 nm due to the aromatic side chains (n → π* transition). However, at this concentration, the weak absorption band of the π → π* transitions at 220 nm was overshadowed by the strong absorption band of the n → π* transitions, which is due to the effect of a polar solvent such as water. Curcumin prepared in a 1 mg/mL ethanol medium showed a maximum absorption band at 484 nm. Curcumin has an enol and keto structure. The absorption band of the keto group, which should be observed at 390 nm, is challenging to observe in ethanol medium, a polar solvent^37^. Therefore, the absorption band of the ethanol form was detected at 484 nm. In the nanocarrier complex, the maximum absorption band of gelatin was detected at 271 nm, while the maximum absorption band of curcumin was shifted to 485 nm at 484 nm. The reason for this shift is due to intermolecular interactions. However, it is not a complete proof whether curcumin is chemically or physically adsorbed on gelatin.

Figure 6e shows the Raman spectra of nanocarriers, gelatin and curcumin. In the Raman spectrum of gelatin, the C-H vibrational mode was detected at 2921 cm^− 1^, the C = O stretching of the peptide bond at 1670 cm^− 1^, the C-C bond vibrations at 1095 cm^− 1^ and 933 cm^− 1^, and the C = C-H_2_ wobble and = C-H bending vibrations at 675 and 555 cm^− 1^^38,39^. However, there was insufficient evidence to annotate the Raman spectrum of curcumin and the nanocarrier complex. Therefore, there is no evidence of chemical binding.

Figure 6f–h shows the DSC analysis of the nanocarrier and other components. According to DSC analysis, the curcumin peaks were detected at 0 °C, 180 °C, and 257 °C. According to literature, the melting peak of curcumin is 184 °C and these results are compatible^40^. A diffuse peak of gelatin was observed at 3 °C and in the range of 194 °C to 203 °C. Melting peaks around 204–206 °C were also reported in the literature^41^. The nanocarrier complex showed a clear, sharp peak at 130 °C. Accordingly, the enthalpy of fusion of curcumin, ΔHm: 464 j/g, and the enthalpy of fusion of gelatin, ΔHm: 1840 j/g, were determined from the device. In contrast, the enthalpy of the nanocarrier complex was determined to be ΔHp: 1346 j/g (Supplementary Material 5). The main question here is whether curcumin binds physically or chemically to the gelatin. In this way, it can be understood whether curcumin is retained in the nanocarrier by chemical or physical adsorption. The literature reports that the glass transition temperatures range from 117 to 147 °C due to interactions between intermolecular hydrogen bonds between lignin and PLA^42^. This increases the possibility of physical adsorption between curcumin and gelatin. Thus, the measured value is the enthalpy of physical adsorption. Furthermore, the thermodynamic enthalpy of the intramolecular O-H covalent bond is 934 kJ/mol, while the enthalpy required for intermolecular O-H. O-H hydrogen bonds are 40.7 kJ/mol. In addition to all these values, which are less than 10–200 kcal/mol, it would be more accurate to speak of dipole-dipole interactions, weak van der Waals interactions and hydrophobic interactions. Even if one converts the units and performs the necessary calculations, 1346 J/g corresponds to an enthalpy of less than 10 kcal/mol. Therefore, it is logical that the drug binds to the carrier via physical adsorption. Therefore, it is correct that no chemical trace was found in the characterization part of the study (Supplementary Material 5).

### The release profile of curcumin from Cur-GelNPs using a membrane

**Fig. 7:**
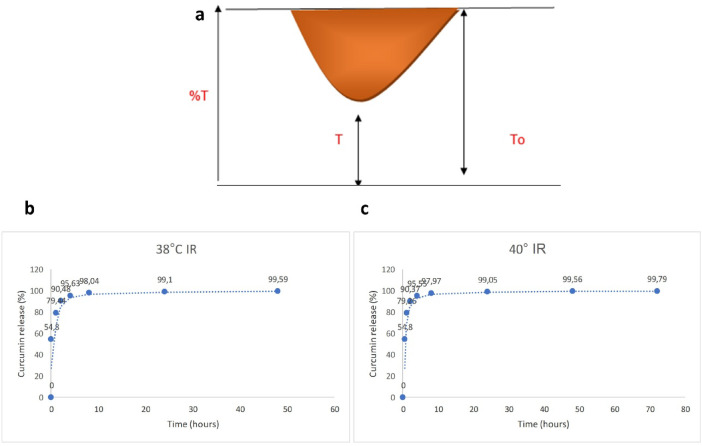
(**a**) NIR spectrum highlighting the characteristic functional groups of Cur-GelNPs. (**b–c**) Curcumin release profiles from Cur-GelNPs at 38 °C (**b**) and 40 °C (**c**), demonstrating temperature-dependent release behavior of the nanocarrier system.

After determining the structural integrity and thermal activation profile of the nanocarrier, cell-based analyses were conducted to assess its biological effects. In general, the infrared spectrum of a prominent functional group in the structure is favored, as shown in the following figure (Fig. 7a). Figure 7a shows the specific vibrational absorption peak of the sample. The ratio of the maximum of this peak to zero and the value of the total absorption peak agree with the equation of Lambert-Beer’s law. The concentration value C can be easily calculated using the formula Abs =- log T = T/To εLC. As a result, the amount of drug incorporated into the solid nanocarrier will decrease or increase the intensity of the absorption peak due to the binding of this functional group. This is because the intensity of the absorption peak decreases when other structures bind to the functional groups in the structure. In this study, the absorption of the carbonyl group, a specific functional group of curcumin, was observed at about 1632 cm^− 1^ (Supplementary Material 6). Using the absorption of a curcumin solution of known concentration as a reference, the concentration of curcumin in the release was calculated using the Lambert-Beer law. Accordingly, at 38 °C and 40 °C, 54.8% of the active substance was released from the membrane in the first half hour. In the first eight hours, 98.04% of the active substance was released at 38 °C and 97.97% at 40 °C. Under NIR light, the release could still be monitored after the first half hour (Fig. 7b and c)

### The penetration of Cur-GelNPs into colon cancer and endothelial cells

The spontaneous fluorescence properties of curcumin were used to detect the uptake of Cur-GelNPs into the cell. Curcumin emits a green-yellow fluorescent glow in the range of 500–550 nm when excited between 420 and 480 nm. Thanks to this property, direct observation with an immunofluorescence microscope was possible without the need for additional staining.

**Fig. 8:**
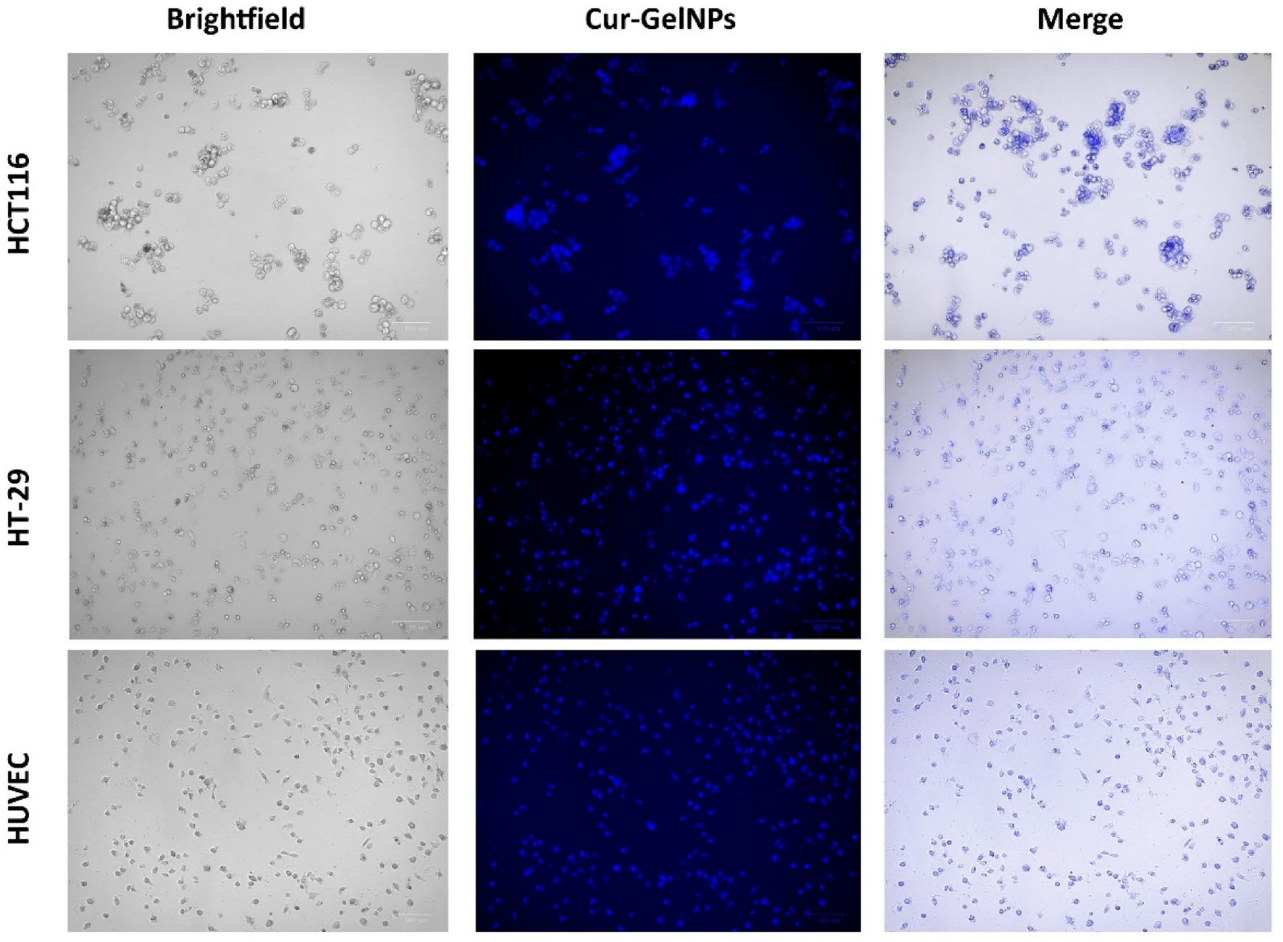
Fluorescence microscopy images showing the intracellular uptake of Cur-GelNPs in treated colorectal cancer cells. The distinct fluorescence signal indicates successful internalization of the curcumin-loaded gelatin nanocarriers, demonstrating their efficient cellular entry and distribution within the cytoplasm.

In our microscopic investigations, green fluorescent signals were clearly observed in cytoplasmic regions of both colon cancer cells (HCT116, HT-29) and endothelial cells (HUVEC). The fact that these signals are localized in intracellular regions and not outside the cell indicates that Cur-GelNPs are internalized within the cell (Fig. 8). Intracellular curcumin showed more intense fluorescence in HCT116 and HT29 colon cancer cells than in normal cells, HUVEC.

### Cytotoxic results in 2D colon cancer cells and 3D co-culture colon cancer model

Statistically significant results were determined using a nonparametric Mann-Whitney test, which involved comparing the HCT116, HT29, and HUVEC control groups with the other groups with and without NIR application at 24, 48, and 72 h. In Figures S1-3, it was found that GelNPs had no effect on cell viability in all treatments, while Cur-loaded nanoparticles decreased cell viability more than Cur-GelNPs without NIR treatment by increasing the release and activity of curcumin by converting to nanocarriers with NIR treatment. The optimal dose of NIR-treated Cur-GelNP was determined to be 25 µg/mL in both colon cancer cell lines (Figs. 2 and 3). NIR-treated Cur-GelNP at a dose of 12.5 µg/mL did not reduce viability below 70% in healthy HUVEC cells, except for 72 h (Figure S3). In HCT116 cells, Cur-GelNP at a dose of 25 µg/mL without NIR reduced cell viability by 69.32% at 24 h, 51.6% at 48 h and 46.97% at 72 h. When NIR was applied, it decreased to 66.45% after 24 h, 42.09% after 48 h, and 24.76% after 72 h (Figure S1). In other colon cancer cells, HT29, Cur-GelNPs at 25 µg/mL without IR decreased cell viability to 80.93% at 24 h, 70.27% at 48 h, and 46.92% at 72 h. In healthy human endothelial cells, HUVECs, Cur-GelNPs at a dose of 25 µg/mL without NIR decreased cell viability to 75.58% at 24 h, 83.19% at 48 h and 47.34% at 72 h. When NIR was applied, cell viability was determined to be 63% after 24 h, 35.93% after 48 h, and 55.82% after 72 h (Figure S3). Detailed results can be found in supplementary material 7. After all analyses were performed, dose-response curves were generated and the IC_50_ values were determined, which are listed in Table 1.

**Table 1:** IC₅₀ values of colorectal cancer cells following treatment with Cur-GelNPs.

IC_50_ (µg/mL)			24 h	48 h	72 h
HCT116	NIR (–)	Cur	32.56 ± 1.513	11.46 ± 1.059	7.013 ± 0.8459
		Cur/GelNPs	21.43 ± 1.331	10.99 ± 1.041	8.133 ± 0.9102
	NIR (+)	Cur	11.82 ± 1.073	7.646 ± 0.8835	6.282 ± 0.7981
		Cur/GelNPs*	**14.46 ± 1.160**	**3.728 ± 0.5715**	**4.185 ± 0.6217**
HT29	NIR (–)	Cur	32.28 ± 1.509	10.59 ± 1.025	8.452 ± 0.9270
		Cur/GelNPs	22.32 ± 1.349	6.150 ± 0.7889	7.419 ± 0.8704
	NIR (+)	Cur	11.94 ± 1.077	8.014 ± 0.9039	6.595 ± 0.8192
		Cur/GelNPs*	**11.32 ± 1.054**	**5.059 ± 0.7040**	**4.524 ± 0.6556**

The dose–response analysis demonstrates the cytotoxic potency of curcumin-loaded gelatin nanocarriers, highlighting differences in sensitivity across cell lines and confirming their therapeutic effectiveness at micromolar concentrations.

*Significant values are in bold.

**Fig. 9:**
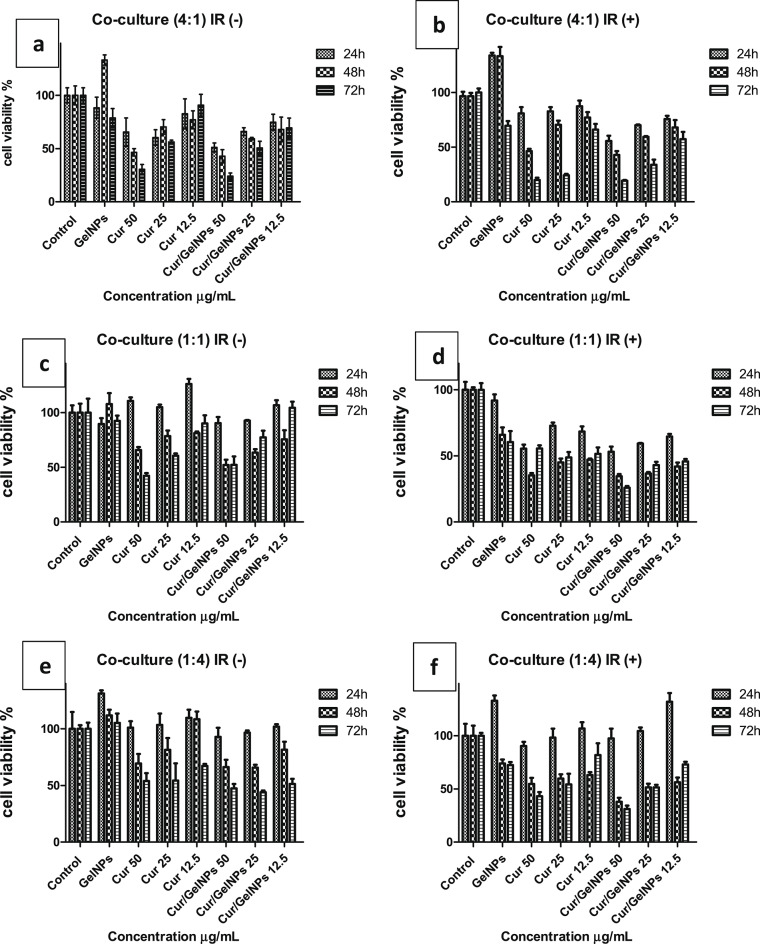
(**a**) NIR-untreated 3D co-culture model (4:1). (**b**) NIR-treated 3D co-culture model (4:1). (**c**) NIR-untreated 3D co-culture model (1:1). (**d**) NIR-treated 3D co-culture model (1:1). (**e**) NIR-untreated 3D co-culture model (1:4). (**f**) NIR-treated 3D co-culture model (1:4). Across all co-culture ratios, analysis of cell viability indicates a clear dose- and time-dependent reduction in metabolic activity following treatment, demonstrating enhanced cytotoxic effects particularly under NIR-exposed conditions.

The efficacy of NIR-treated Cur-GelNPs on cell proliferation was also investigated by creating a co-culture model with colon cancer and healthy endothelial cells in different ratios (4:1, 1:1, 4:4). Accordingly, NIR application in the co-culture model formed by seeding colon cancer cells and healthy endothelial cells in a ratio of (4:1), especially in the lowest dose of 12.5 µg/mL Cur-GelNP treated groups, reduced the viability rate from 68% to 53.6% at 48 h, while the rate at 24 h was similar (75%). After 72 h, this rate was also 12% lower (Fig. 9a, b). This result is similar to Figs. S1 and S2, in that the Cur-GelNP-treated groups in HCT116 and HT29 colon cancer cells showed a decrease in viability of 5–28%, especially in the NIR-treated groups at 48 h. Figure 9c and d show that NIR treatment in the 1:1 co-culture model reduced the viability of Cur-GelNPs at a dose of 12.5 µg/mL from 106.73 to 64.7% at 24 h and from 75.58 to 41.91% at 48 h. This difference persisted after 72 h. In the 1:4 co-culture model with a small number of colorectal cancer cells, the viability of Cur-GelNPs at a dose of 12.5 µg/ml did not change with NIR treatment after 24 h; after 48 h, the main activity was 25% lower than in the non-NIR-treated group; after 72 h, the viability remained at 72.94% and decreased to 51.42% in the non-NIR-treated group (Fig. 9e, f).

### Results of the 2D wound healing and 3D transwell^®^ invasion/migration tests

**Fig. 10:**
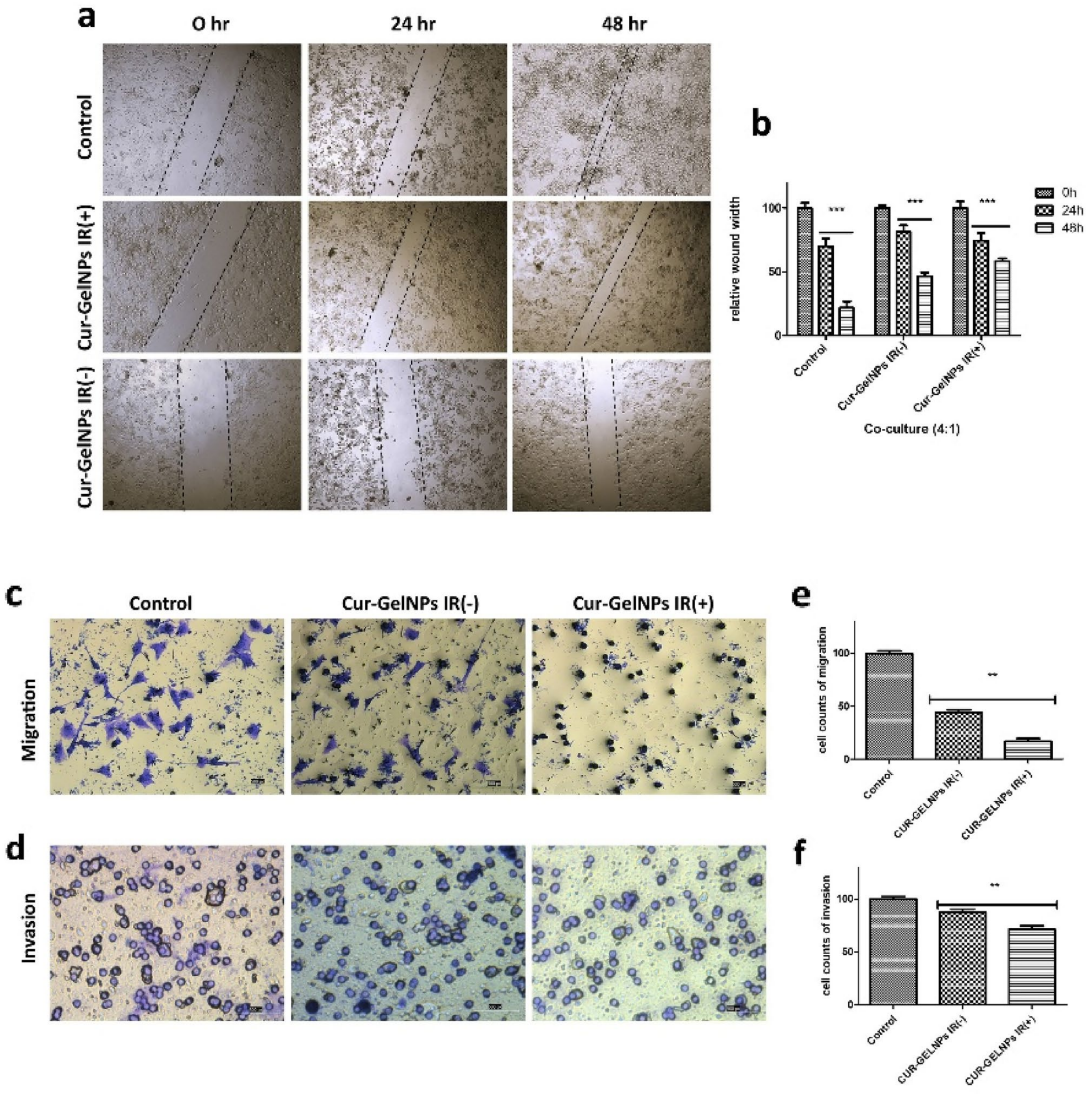
(**a**, **b**) Wound closure test in 3D co-culture (4:1) treated or untreated Cur-GelNPs groups with and without NIR treatment. Transwell assay to evaluate the migration and invasion ability of the co-culture (4:1) model. (**c**, **e**) Migration ability of the IR-treated Cur-GelNPs. (**d**, **f**) Invasion ability of NIR-treated Cur-GelNPs. Data are presented as mean ± SEM, *n* = 3, **p < 0.05. Scale bar = 50 μm.

When the results of wound closure were evaluated, a wound closure rate of 85% was observed in the control group at the end of the 48th hour, while this rate was 60% in the groups where only Cur-GelNPs were applied. In the groups where Cur-GelNP was applied with NIR, this rate remained at 42 (Fig. 10a).

Transwell analysis was performed using a colon cancer model prepared in a 4:1 ratio in a 3D co-culture. After seeding, the cells were treated with Cur-GelNPs at an IC_50_ ratio, and NIR was applied. According to the data after 24 h, NIR treatment decreased cell migration by 28% and invasion by 16% compared to the non-NIR-treated group (Fig. 10b, c).

### Results of apoptosis of colon cancer cells

**Fig. 11:**
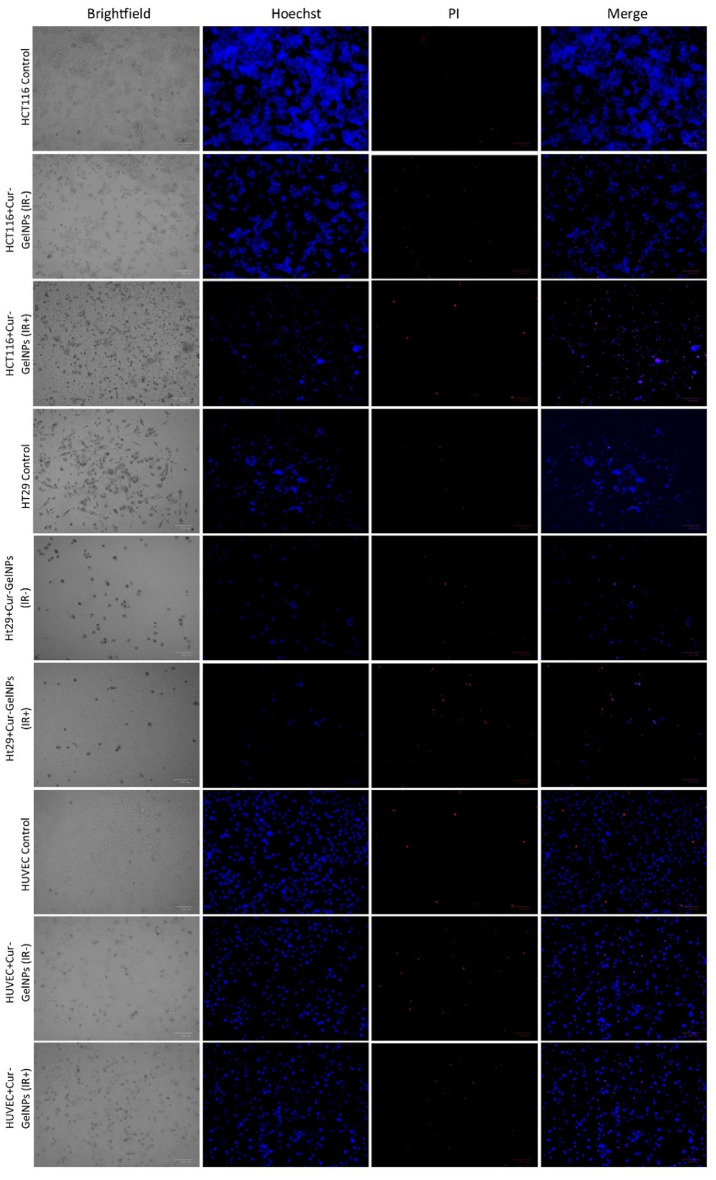
Detection of apoptotic cells by Hoechst/PI double staining following NIR exposure in colon cancer cells (HCT116 and HT-29) and healthy endothelial cells treated with Cur-GelNPs at their respective IC₅₀ concentrations. The nuclear condensation and membrane-compromised cells are visualized to assess treatment-induced apoptotic responses. (Scale bar = 100 nm).

Figure 11, NIR treatment of Cur-GelNP groups of colon cancer cells HCT116 and HT29, shows the nuclear changes in the cells as a result of double staining with Hoechst33342 and PI. Hoechst33342 binds to the nuclear DNA of the cells. Based on microscopic findings after 24 h of IC_50_ doses, NIR treatment was found to induce significant apoptosis in the treated groups. The composite images show early apoptosis (light pink) and late apoptosis/necrosis (dark pink). In the HCT116 + Cur-GelNP and HT29 + Cur-GelNP cell lines with IR treatment, late apoptotic/necrotic cells were more intense compared to those without NIR treatment. In contrast, the apoptotic cell ratio in healthy HUVEC cell lines was similar to that in the control group (Fig. 11).

### Evaluation of mitochondrial membrane potential in colorectal cancer cells treated or not treated with IR

**Fig. 12:**
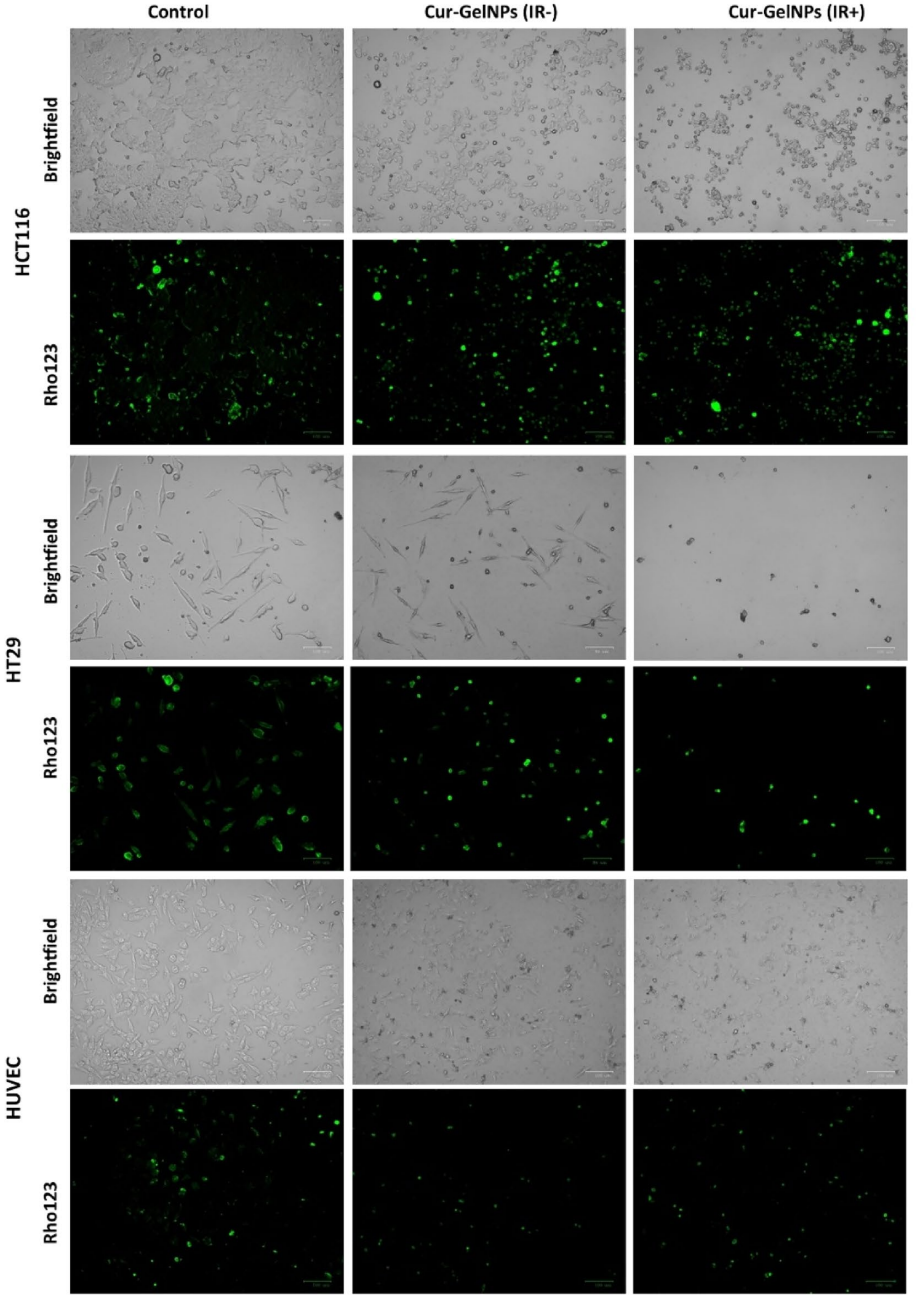
Detection of NIR-induced mitochondrial activity in colon cancer cells (HCT116 and HT-29) and healthy endothelial cells treated with Cur-GelNPs at their respective IC₅₀ concentrations, visualized by Rho123 staining. Changes in mitochondrial membrane potential following treatment are highlighted by variations in fluorescence intensity (Scale bar = 100 nm).

Mitochondrial membrane potential, phosphorylation and ATP decrease as a result of increased mitochondrial permeability and membrane pores. Apoptosis is a process that causes cell shrinkage and condensation of its organelles, eventually leading to cell lysis. Mitochondrial shrinkage is a hallmark of late apoptosis. The mitochondrial membrane potential can be measured by staining with Rho123. Green fluorescence is detected in cells with high membrane potential. NIR treatment of the HCT116 and HT29 Cur-GelNP cell lines resulted in a lower mitochondrial membrane potential compared to the cells without NIR treatment. HUVEC cells, on the other hand, appear to have a similar mitochondrial membrane potential in all groups (Fig. 12).

**Fig. 13:**
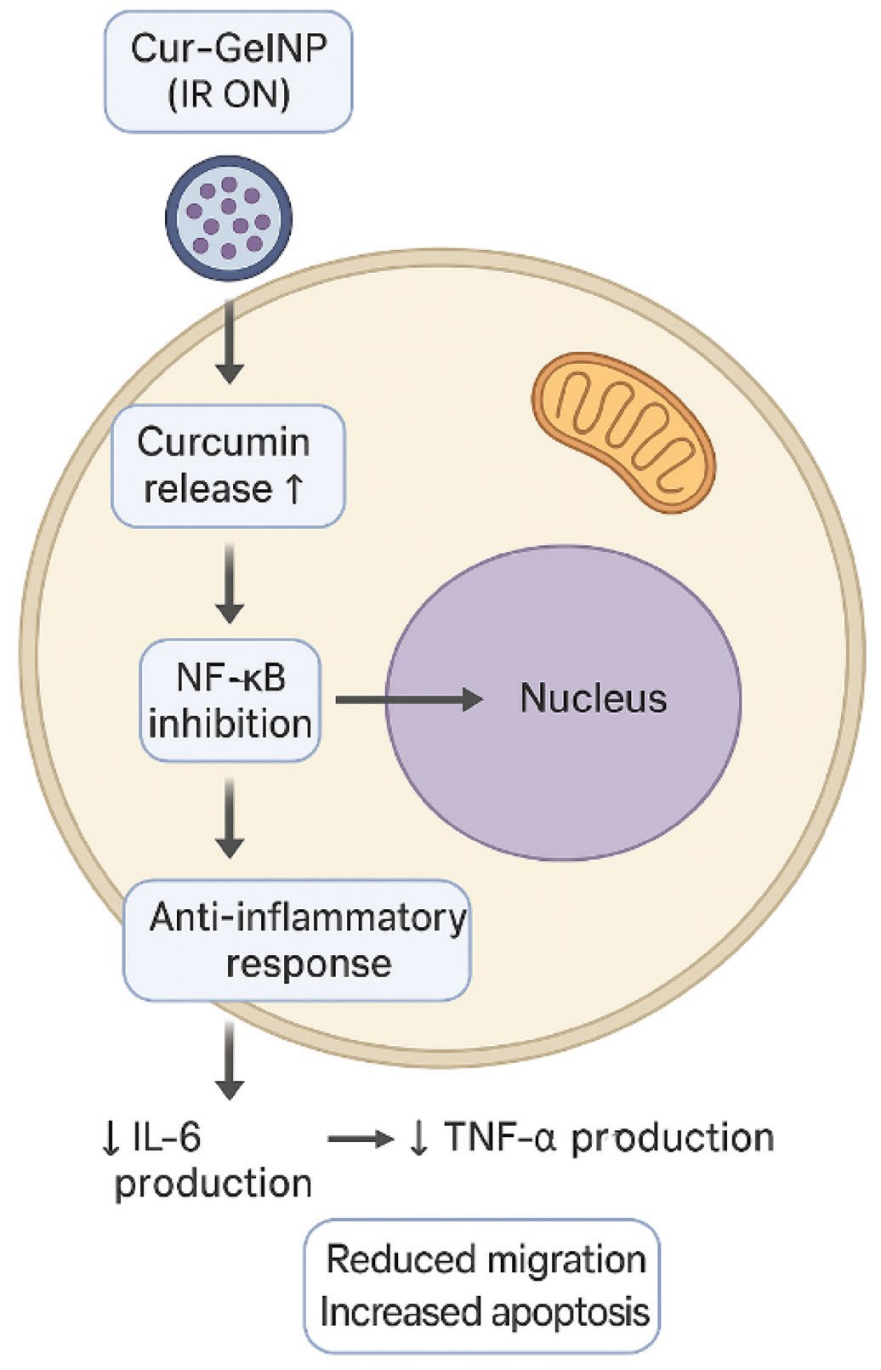
Proposed mechanism of antioxidant and anti-inflammatory action of NIR-activated Cur-GelNPs. This schematic was created using BioRender (BioRender.com; https://www.biorender.com; version accessed in 2025).

Figure 13 is a schematic illustration summarizing the proposed antioxidant and anti-inflammatory effects of NIR-activated Cur-GelNPs. Curcumin release from the nanoparticles reduces intracellular reactive oxygen species (ROS), which in turn prevents NF-κB nuclear translocation and lowers pro-inflammatory cytokines IL-6 and TNF-α, consistent with findings reported in the literature^43–45^. This figure is intended for illustrative purposes and does not present original experimental data from this study.

## Discussion

The nanocarriers already known in the literature are not suitable for cancer drug studies due to several limitations, including complex and costly production, stability issues, potential toxicity and immunogenicity, limited targeting efficiency, unpredictable drug-release profiles, and significant regulatory and safety challenges^46^. For this reason, we used gelatin to exhibit temperature-sensitive behavior, and under different thermal conditions, the active ingredients loaded into the gelatin affect the release profile^20^.

The application of curcumin in the food industry and in pharmaceutical formulations is limited due to its poor solubility and low stability under various conditions^47^. Curcumin is very sensitive to light, heat treatment, alkaline conditions, oxygen, enzymes, metallic ions and ascorbic acid. To overcome these limitations, nano-capsulation is considered suitable^48^. Another unknown benefit of curcumin is its effectiveness. Namely, that they emit fluorescent light. Thanks to these properties, the cell can be labeled in such studies with small molecules that emit fluorescence and can be tracked inside the cell^49^. Moreover, when curcumin interacts with gelatin, it surrounds the gelatin molecule and can easily bind to the hydrophobic pockets of gelatin^21^. To overcome these obstacles, curcumin-loaded gelatin-based nanoparticles (Cur-GelNPs) were developed to enhance the cellular uptake of curcumin.

Firstly, HPLC is used to determine the curcumin loading capacity in gelatin nanocarriers accurately. According to the HPLC results, the highest amount of curcumin was captured by 30 s of sonication, resulting in a 64% yield in gelatin, which serves as a nanocarrier. Although a 10% curcumin/carrier ratio appears to be the most adsorbed curcumin amount, this amount may not be sufficient for the curcumin’s effect on cancer in later cancer tests. Therefore, the next highest amount, a 1:1 curcumin/carrier ratio, and 30 s of sonication parameters were selected as the optimum parameters. In addition to these, zeta potential − 7.89 mV is one of the lowest values in a 1:1 curcumin/carrier ratio and 30 s sonication parameters. It has been previously reported in the literature that nano-drugs with a negative surface charge are more effective in cancerous areas that form an acidic zone^50,51^.

Secondly, to assess the stability of Cur-GelNPs, zeta potential measurements were used. The lower the zeta potential, the lower the conductivity of the solution. This is because the surface charge of the particle is also a measure of the particle’s ability to move in the solution at a certain potential difference. If the surface charge is low, the mobility also decreases. However, a high negative surface charge is sufficient for our purposes. This is because this nanocarrier is designed to be released in the Krebs area, thanks to the infrared light source, and not under the influence of an electric current. It is also essential that the Cur-GelNPs have a size of 24, 30, and 40 nm at different temperatures, because nanoparticles of this size easily penetrate cells. This is because nanoparticles cannot enter the cell due to increased permeability and the retention effect (EPR) at sizes above 200 nm, which falls within the cancer range^52^. In another study, NIR light with an energy of 150 W was used as a thermal and vibrational energy source to synthesize gelatin-based carboplatin/gelatin nanoparticles (CP-NPs) of different sizes. Initially, gelatin-based CP-NPs with sizes of 10 nm and 24 nm were obtained at 30 and 35 °C, respectively. Interestingly, the size of the NPs increased to 190 nm at 40 °C and decreased to 15 nm and 16 nm at 45 and 50 °C, respectively. Gelatin-based CP-NPs with different properties and sizes were formed at different temperatures when heated with NIR light. CP-NPs-50, synthesized at 50 °C, were found to be more stable than the others (PDI = 0.607)^20^. If the PDI is greater than 0.725, the particles are very unstable. In other words, although the particle size is in the nanometer range, one cannot speak of a stable nanoparticle. In our study, a sonication time of 30 s (PDI = 0.522) with the smallest particle size of 24 nm was selected as the optimal parameter. Then, when the PDI values were compared at different temperatures (37–38 °C-39–40 °C-41–42 °C), the stability of Cur-GelNP began to deteriorate as Cur-GelNPs swelled under the effect of heat. Therefore, 38 °C proved to be the most suitable parameter for the activation of the nanocarrier and the release of Cur-GelNPs with infrared light. Moreover, the Cur-GelNPs released by near infrared light were activated very rapidly and released 1.35 times more curcumin than the non-activated nanocarrier in the first half hour. In addition, the Cur-GelNPs released by near infrared light were activated very quickly and released 1.35 times more curcumin in the first half hour than the non-activated nanocarrier. It was also observed that the release under NIR light could still be controlled after the first half hour. Danışman-Kalındemirtaş et al. 2022^20^, 94% CP was released from CP-NPs-50, 93% from CP-NPs-40 and 81% from CP-NPs-30 at the end of 20 h. In our study, the rate was above 97% in the first 10 h at both 38 °C and 40 °C.

The fluorescence absorption value of curcumin is 427, 430 nm^53^. Thirdly, we used curcumin due to its fluorescent light emission; the Cur-GelNPs were easily visible under an immunofluorescence microscope and localized within the cell, particularly in the cytoplasm. In one study, gelatin-functionalized magnetic Fe₃O₄ nanoparticles (gel MNPs) were prepared using a simple desolation and co-precipitation method. The gelatin coating enabled the loading of curcumin through hydrophobic interaction. CUR-GEL MNPs were found to be present in both the cytoplasm and nucleus of A-549 (lung) and MCF-7 (breast) cancer cells when exposed to heat under an alternating magnetic field (ACMF), similar to our study^54^.

Fourthly, due to the thermo-reversible gel formation of gelatin, the gelatin-based NPs can be converted into nanocarriers by the variable thermal effect of the IR lamp to deliver curcumin from the Cur-GelNPs to the target site. Some special nanoparticles can convert light energy into heat and destroy tumor cells^55^. In a study, polyethylene glycol-curcumin-gold nanoparticles (PEG-Cur-Au NPs), a novel type of nanoparticles with photothermal properties, were synthesized, characterized, and applied in photothermal treatment (PTT) of melanoma cancer cells, showing a cytotoxic effect at concentrations of 25 µg/mL and above. The IC₅₀ was determined to be 42.7 µg/mL. No toxicity was observed below 10 µg/mL. When the laser was applied in conjunction with PEG-Cur-Au NPs, a strong PTT effect was observed, even at very low concentrations^56^. In another similar study, implantable thermo-sensitive drug-loaded magnetic nanofibers (NFs) have attracted significant interest for localized thermo-chemotherapy of cancer tissue/cells. From this perspective, smart polymeric electrospun nanofibers were developed that incorporate magnetic nanoparticles (MNPs) and curcumin (CUR), a natural polyphenolic anticancer agent, to enhance local hyperthermic chemotherapy against the most severe form of skin cancer, melanoma. The CUR/MNPs-loaded thermo-sensitive electro-spun nanofibers exhibited heat generation sensitivity to an alternating magnetic field (AMF) and a “ON-OFF” switchable heating capability. Moreover, due to reversible changes in swelling rate, CUR release from magnetic nanofibers was also found to be switchable in response to the “ON-OFF” transition of AMF application. Due to the combinatorial effect of hyperthermia and CUR release after applying AMF (“ON” state) for 600 s on the second and third incubation days, the viability of B16F10 melanoma cancer cells exposed to CUR/MNPs-NFs decreased by 40% and 17%, respectively^57^. In a separate study, the effect of curcumin-containing carboxymethyl chitosan coated with iron (II, III) oxide (Fe₃O₄) nanoparticles on breast cancer cells (MCF-7 and MDA-MB-231) and human fibroblasts, combined with hyperthermia, was investigated. MNP-CMC-CUR (magnetic nanoparticles + carboxymethyl chitosan + curcumin) was synthesized and characterized. The hyperthermia system was constructed using a 210-turn copper wire coil and a high-voltage power supply. Cells were incubated at MNP-CMC-CUR concentrations of 10–40 µg/mL for 30 min at 60 °C and 48 h at 43 °C. The combined treatment significantly reduced the metabolic activity of MCF-7 cells compared to monotherapy^58^. However, the temperatures applied here are not applicable in vivo. Therefore, in our system, a temperature of 38 °C can be achieved with NIR application in a short time, such as 30 s, and the Cur-GelNP dose shows a cytotoxic effect at 25 µg/mL in both colon cancer cell lines. We used free curcumin for the positive control. When the cell viability of HCT116 was found to be 53.30% with free curcumin and 51.6% with Cur-GelNPs at 25 µg/mL after 48 h. With NIR exposure, the colon cancer cell viability decreased to 51.64% for free curcumin and 42.09% for Cur-GelNPs. Without NIR treatment, the cell viability of HT-29 was observed as 71.49% for free curcumin and 70.27% for Cur-GelNPs. When NIR was exposed to the cells, the viability of HT-29 declined to 49.95% for free curcumin and 35.93% for Cur-GelNPs (Supp. Mat. 7., FigureS1). In this case, while the effect of curcumin loaded into the gelatin nanocarrier system was similar in both colorectal cancer cell lines without NIR treatment, when NIR was applied, Cur-GelNPs at the same dose were observed to be more effective in both cancer cell lines, particularly in HT-29 cells. Thus, these carrier systems can exhibit targeted cytotoxic effects, such as those induced by NIR, at lower doses. This rate was even higher compared to Cur-GelNPs without NIR, with an IC_50_ value of 15.42 µg/mL at 48 h, whereas in HT-29, it was 7.878 µg/mL. This dose did not reduce the viability of healthy HUVEC cells to less than 70%. In this study, a co-culture model was also established using colon/endothelial cells in different ratios to create a tumor microenvironment. In the co-culture model (4:1), in which colon cancer cells were overrepresented, Cur-GelNPs, whose activity increased with NIR application, exhibited a cytotoxic effect. At the same time, cell migration and invasion were significantly reduced compared to both the control group and the Cur-GelNPs co-culture model without NIR application. This proves that IR treatment enhances the release and activity of curcumin from gelatin nanoparticles.

Finally, we found that NIR-treated Cur-GelNPs increased apoptosis and mitochondrial dysfunction. Mitochondrial membrane potential, phosphorylation and ATP are decreased as a result of increased mitochondrial permeability and membrane pores. Given that mitochondrial shrinkage is a well-established indicator of late-stage apoptosis^59^, our results clearly support the activation of intrinsic apoptotic pathways. Overall, these outcomes are consistent with our experimental observations and confirm the potential of IR-responsive Cur-GelNPs as an effective strategy for targeted cancer therapy.

## Conclusions

In this study, we successfully developed curcumin-loaded gelatin-based controlled drug delivery nanocarriers that are NIR-responsive and exhibit rapid and controlled release in a 3D co-culture model of colon cancer and endothelial cells. When the characterization, release and biological activity data obtained in the study were evaluated together, the following conclusions were reached. Remarkably, increasing the temperature to 38 °C for only 30 s resulted in significantly increased curcumin release from GelNPs exposed to NIR, compared to GelNPs without NIR treatment. In addition, the co-culture significantly reduced cell viability, invasion and migration of colon cancer cells. In addition, apoptosis and mitochondrial dysfunction were induced. Considering that cell proliferation in healthy endothelial cells and in the co-culture model (1:4) was not very effective, this suggests that this method may be applicable in the target area of chemotherapy. Nevertheless, further in vivo preclinical and clinical studies are essential to comprehensively assess the long-term safety and therapeutic efficacy of this approach.

The Cur-GelNP system is a unique therapeutic platform due to its NIR-triggered release mechanism, low-energy production method through spontaneous adsorption, temperature-sensitive stability profile, and demonstrated biological activity in a 3D co-culture model. It differs from the gelatin-curcumin nanoformulations previously discussed.

## Supplementary Information

Below is the link to the electronic supplementary material.

Supplementary Material 1

Supplementary Material 2

Supplementary Material 3

Supplementary Material 4

Supplementary Material 5

Supplementary Material 6

Supplementary Material 7

## Data Availability

Data supporting the findings of this study are available in the Supplementary Materials accompanying this article. Additional data relating to this study can be obtained from the corresponding author upon reasonable request.
