# Supplementary material for: Synthesis and characterization of NIR-sensitive curcumin-gelatin nanoparticles for targeted drug delivery in 3D colon cancer
Source: Sci Rep. 2026 Mar 5;16:12167. doi: 10.1038/s41598-026-42199-3 (PMC13076676; doi:10.1038/s41598-026-42199-3)
Supplement: Supplementary file 1 — Supplementary Material 1 [file 41598_2026_42199_MOESM1_ESM.docx]

**Supplementary Material 1 for:**

**Synthesis and Characterization of NIR-Sensitive Curcumin-Gelatin Nanoparticles for Targeted Drug Delivery in 3D Colon Cancer**

Dilşad Özerkan^1*^, Ferdane Danışman-Kalındemirtaş^2*^, İshak Afşin Kariper^3^

^1*^ Kastamonu University, Faculty of Engineering and Architecture, Department of Genetic and Bioengineering, Kastamonu/TURKEY

^2*^Erzincan Binali Yıldırım University, Faculty of Medicine, Department of Physiology, Erzincan, TURKEY

^3^ Erciyes University, Education Faculty, Department of Science Education, Kayseri, TURKEY

**DLS-ZETA**

**SONICATION:**

**Gelatin: PDI: 0.416, Kcps: 181.3, Z: 178 nm, Avg: 20.54 nm**

**
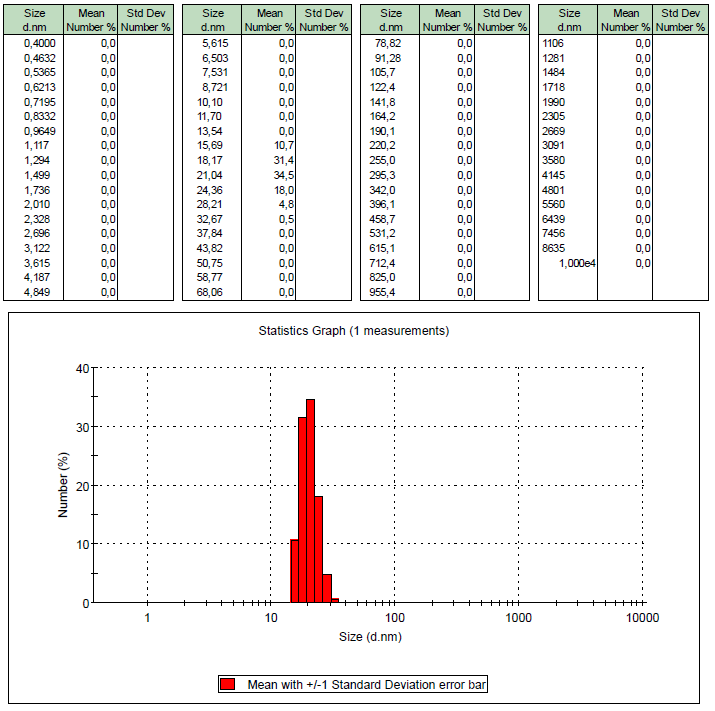
**

**
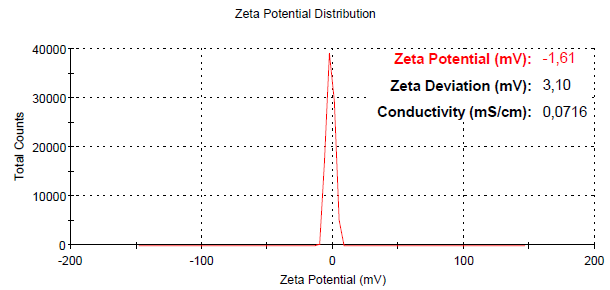
**

**Curcumin: PDI: 0.084, Kcps: 808, Z: 643 nm, Avg: 551.54 nm**

**
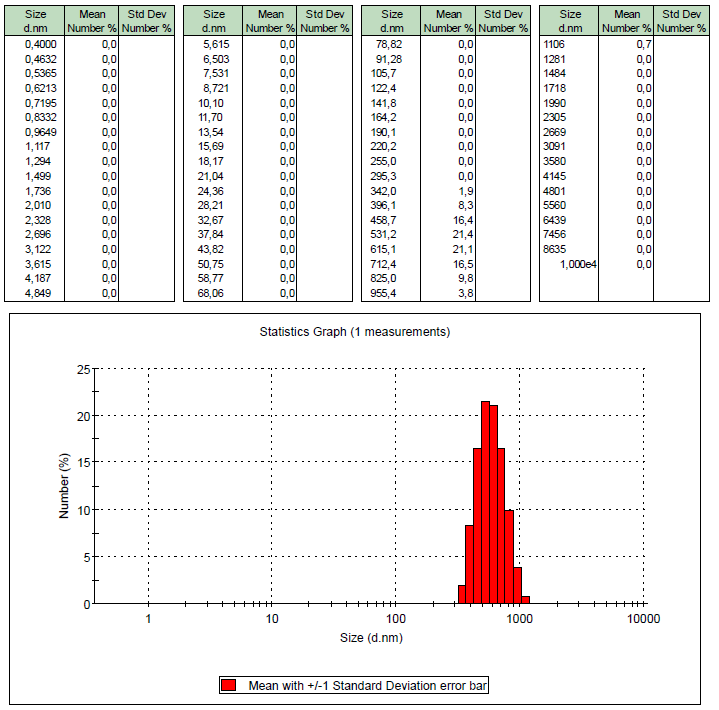
**

**
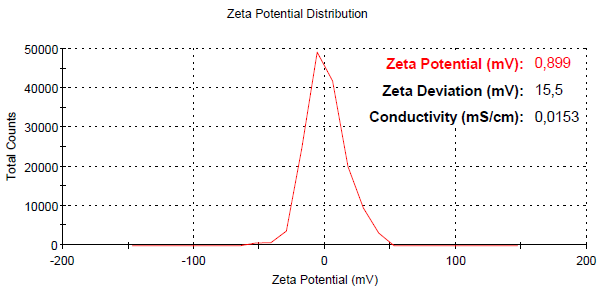
**

**1 s Sonication, PDI: 0.518, Kcps: 90, Z: 1434 nm, Avg: 51.51 nm,
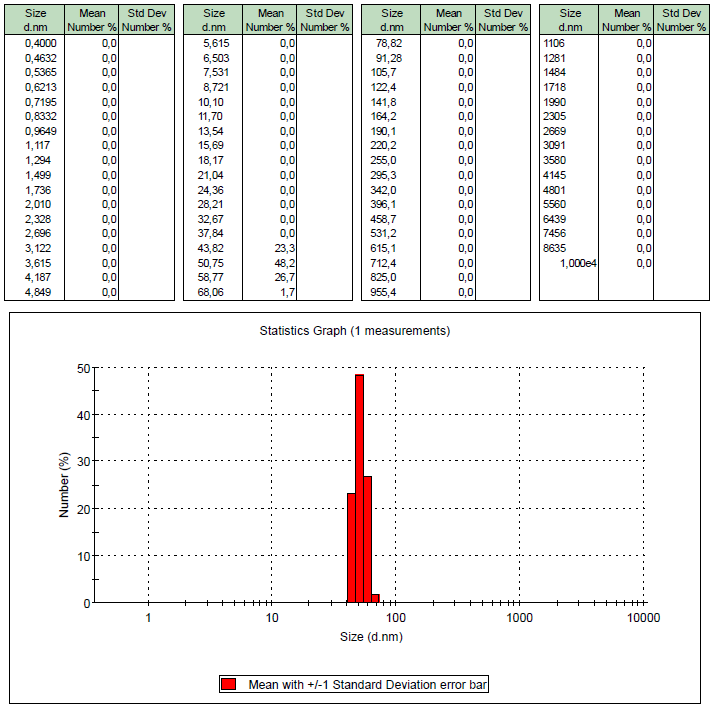
**

**
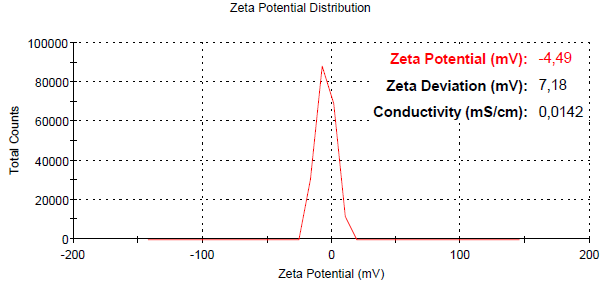
**

**5 s Sonication, PDI: 0.505, Kcps: 46, Z: 1555 nm, Avg: 31.58 nm,
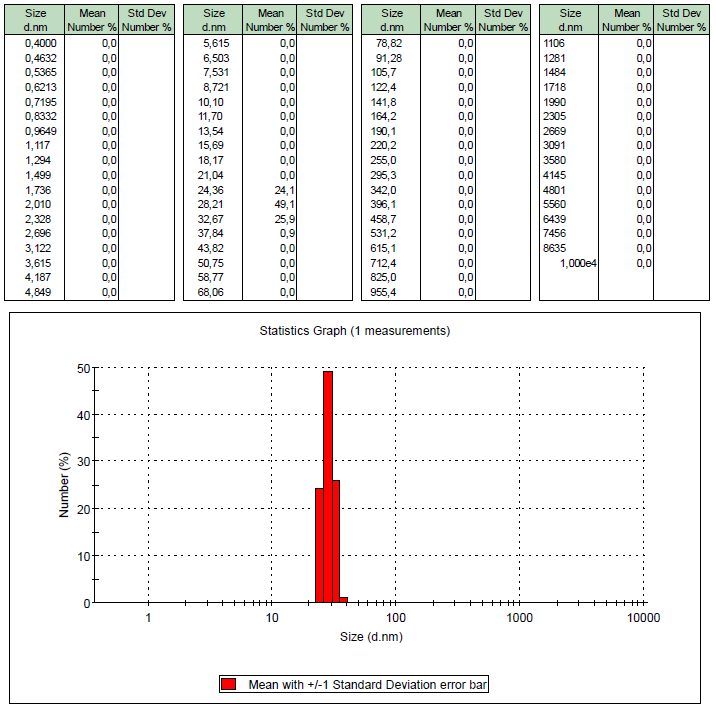
**

**
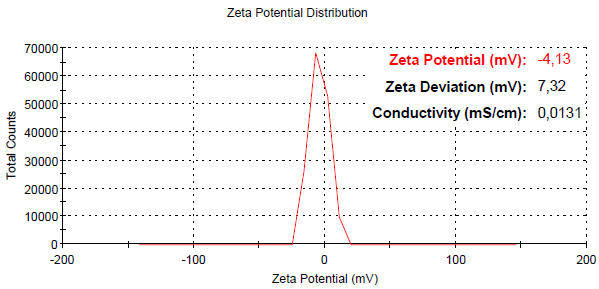
**

**10 s Sonication, PDI: 0.502, Kcps: 38, Z: 1360 nm, Avg: 41. 35 nm**

**
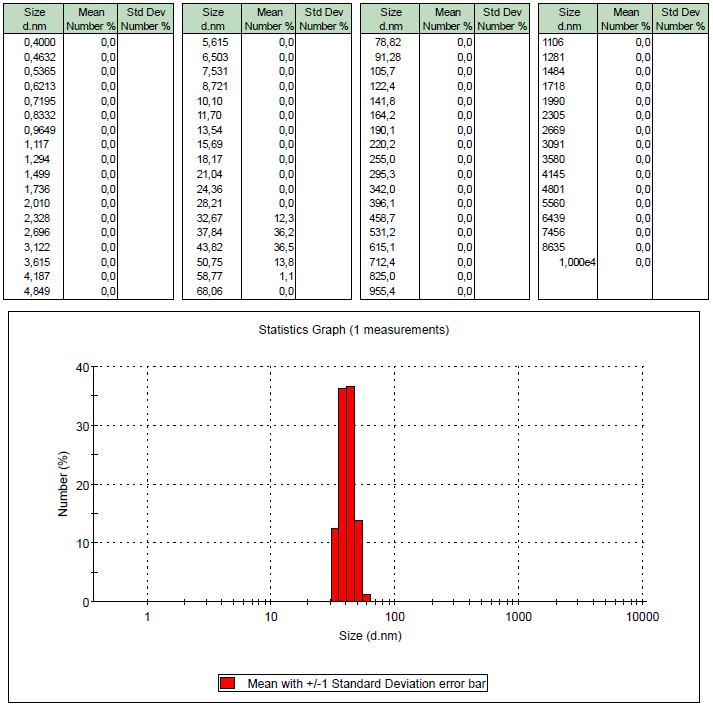
**

**
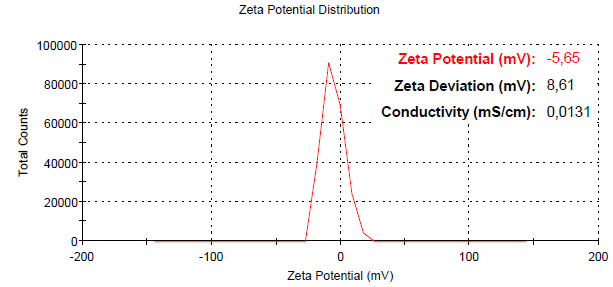
**

**30 s Sonication, PDI: 0.522, Kcps: 51, Z: 1514 nm, Avg: 24.84 nm,**

**
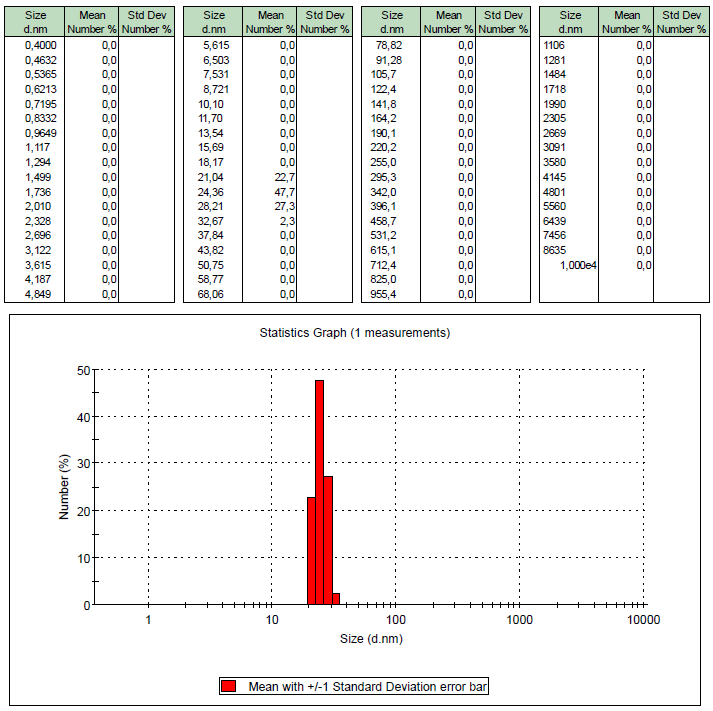
**

**
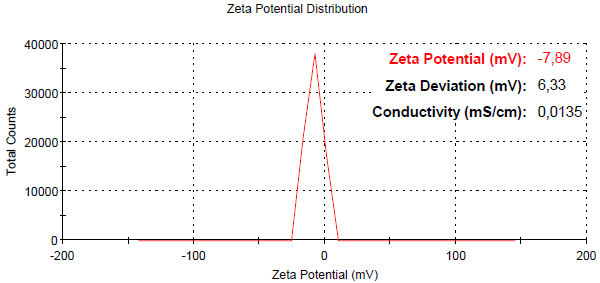
**

**60 s Sonication, PDI: 0.585, Kcps: 50, Z: 1885 nm, Avg: 38.47 nm, A
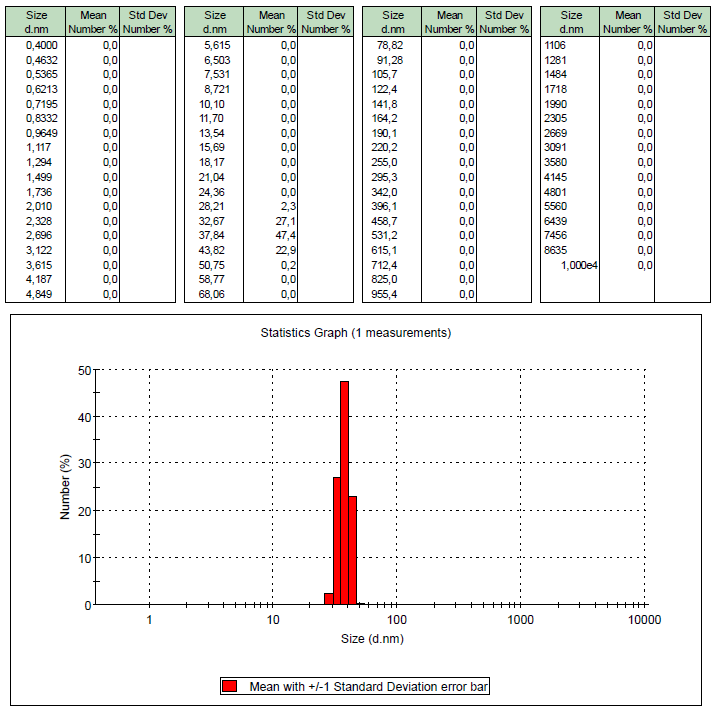
**

**
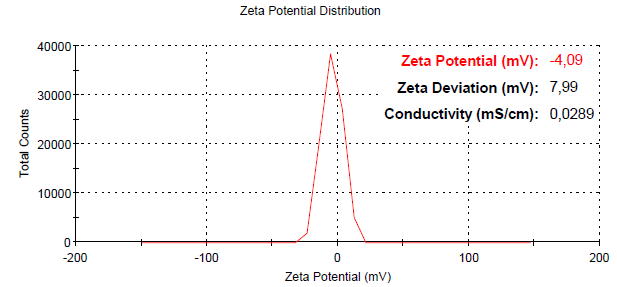
**

**120 s Sonication, PDI: 0.380, Kcps: 44, Z: 1684 nm, Avg: 58.67 nm,
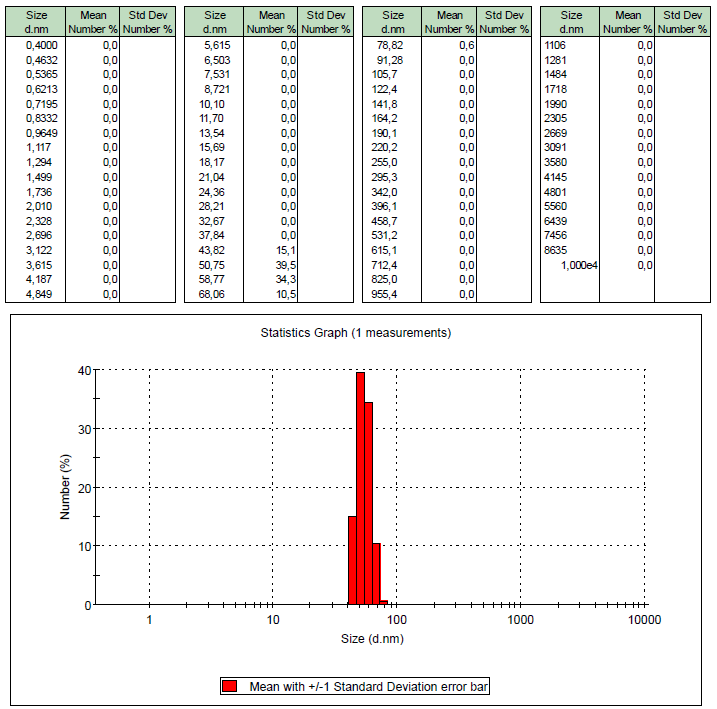
**

**
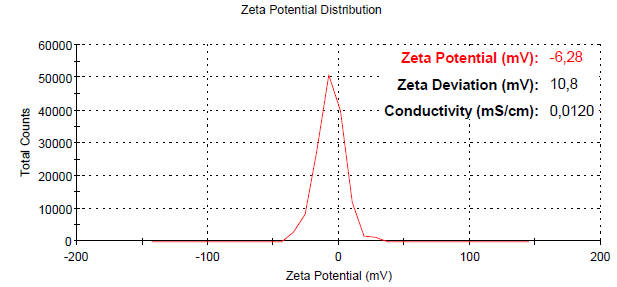
**

**CONCENTRATION**

**0.1 ratio Curcumin/Gelatin (w/w)**

**PDI: 0.185, Kcps: 7710, Z: 157 nm, Avg: 99.94 nm,
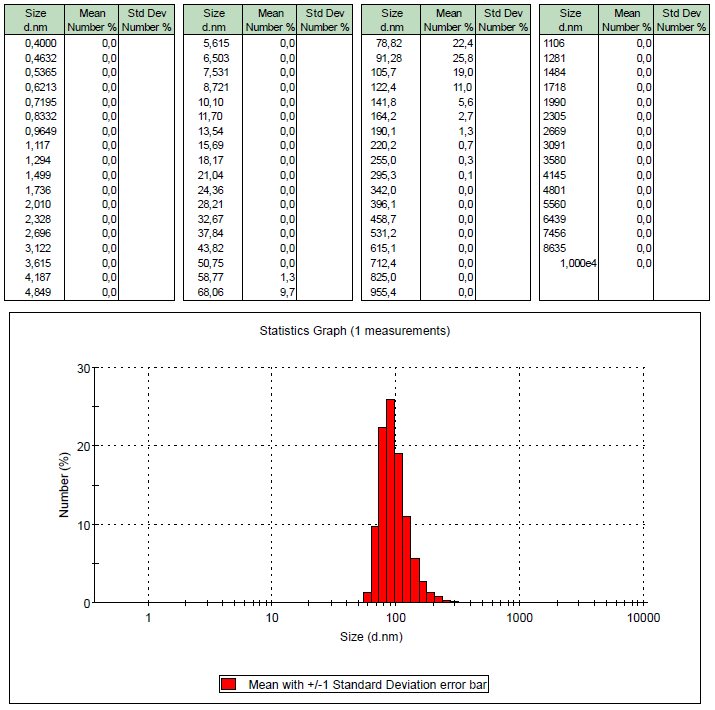
**

**
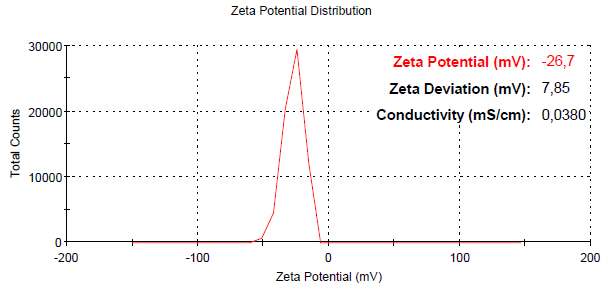
**

**0.25 ratio Curcumin/Gelatin (w/w)**

**PDI: 0.199, Kcps: 8742, Z: 250 nm, Avg: 67.42 nm,**


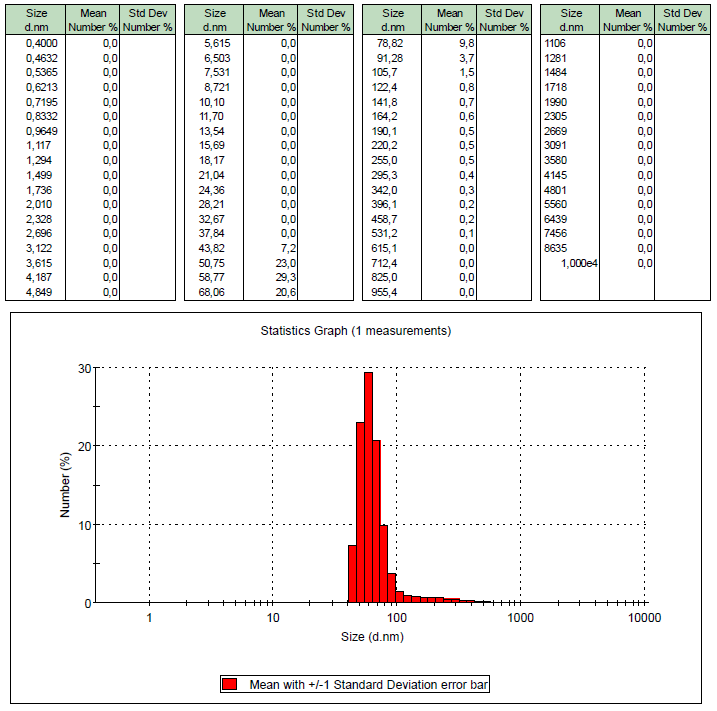


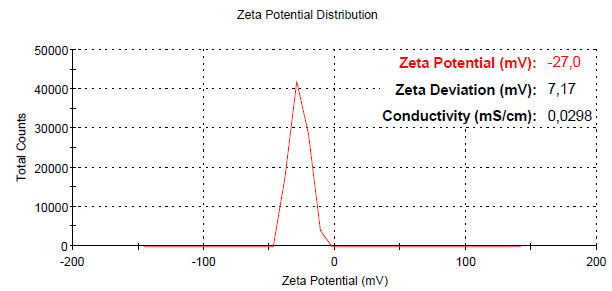


**0.50 ratio Curcumin/Gelatin (w/w)**

**PDI: 0.624, Kcps: 53.21, Z: 1466 nm, Avg: 812.90 nm,**

**
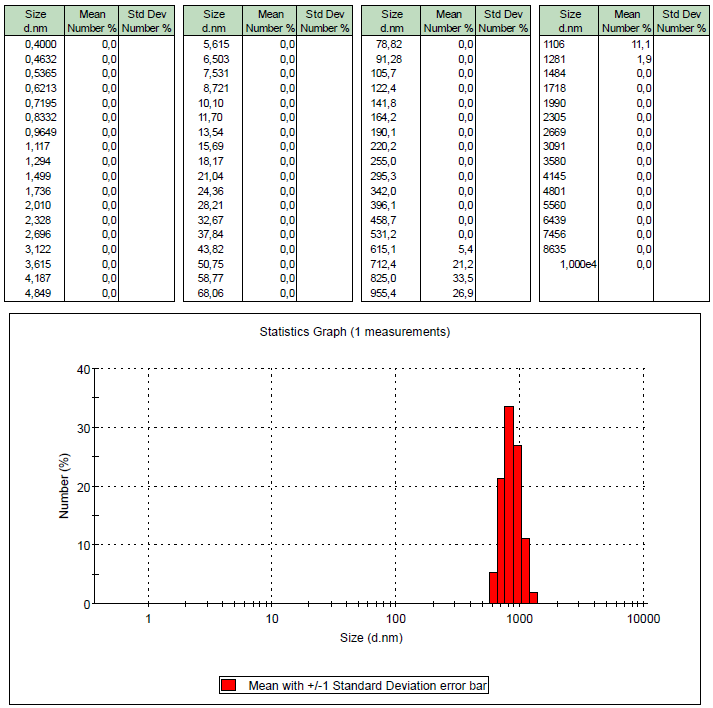
**

**
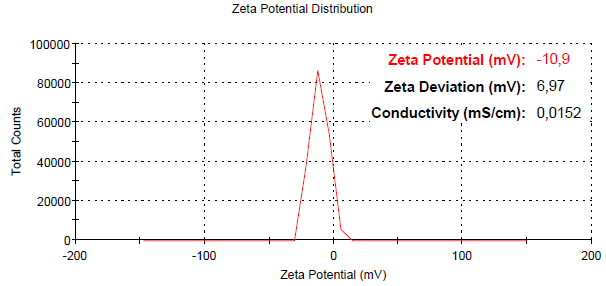
**

**0.75 ratio Curcumin/Gelatin (w/w)**

**PDI: 0.407, Kcps: 137, Z: 880 nm, Avg: 180.69 nm,**

**
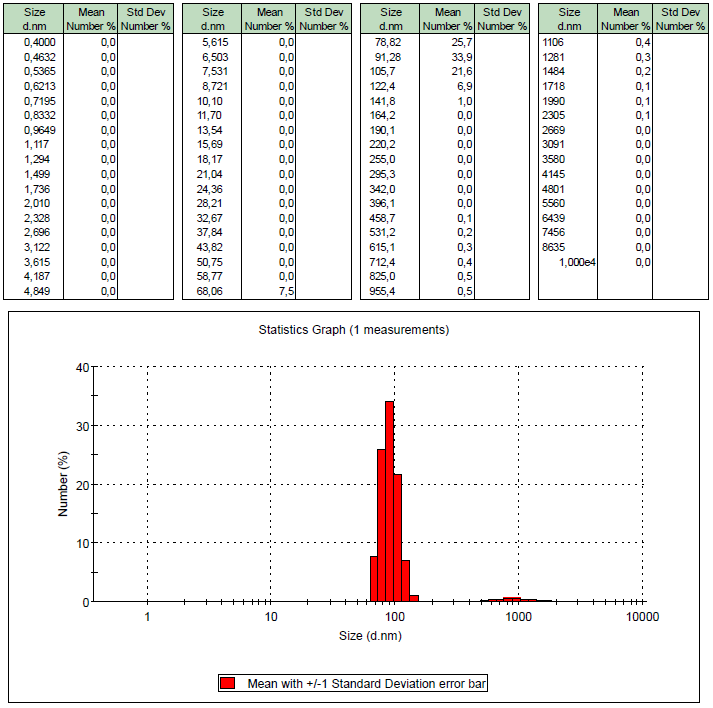
**


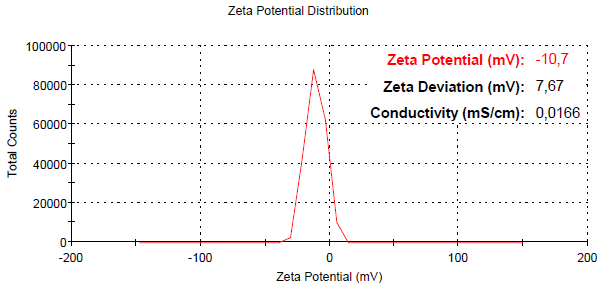


**1 ratio Curcumin/Gelatin (w/w)**

**PDI: 0.502, Kcps: 38, Z: 1360 nm, Avg: 41. 35 nm**

**
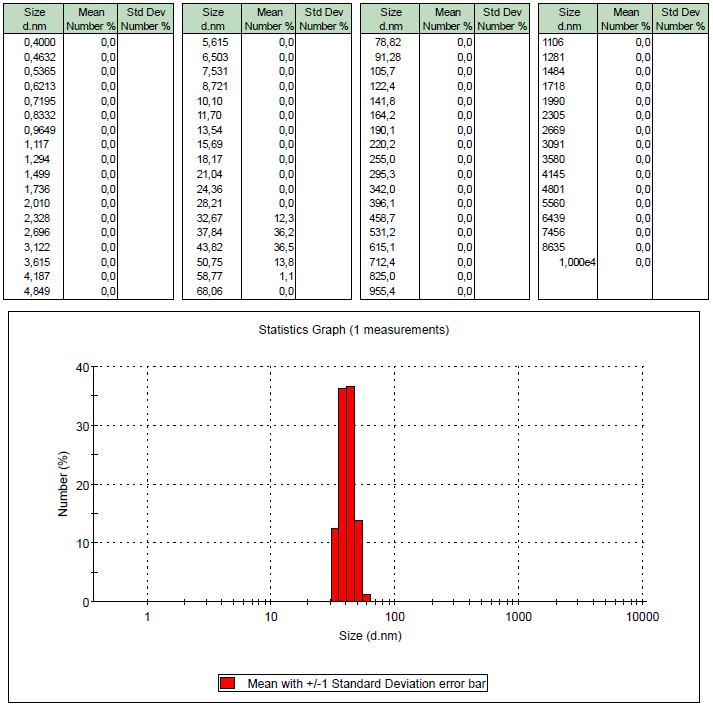
**

**
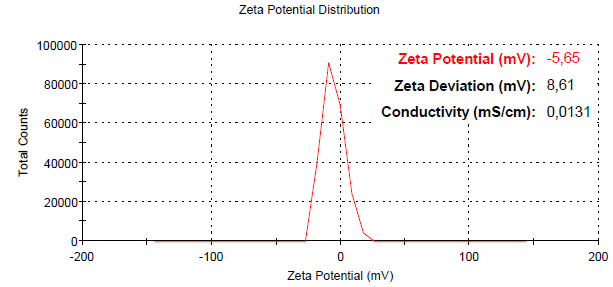
**

**1.25 ratio Curcumin/Gelatin (w/w)**

**PDI: 0.993, Kcps: 43, Z: 883 nm, Avg: 37.01 nm**

**
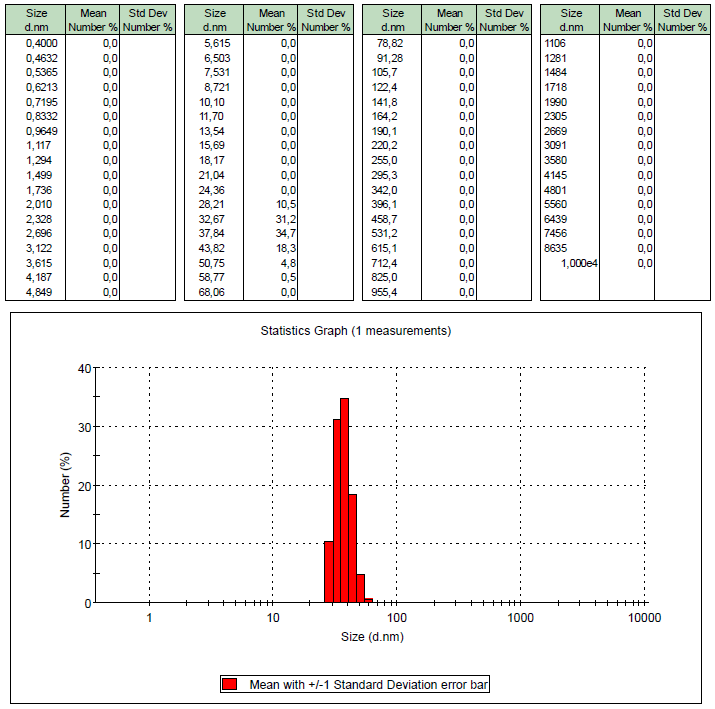
**

**
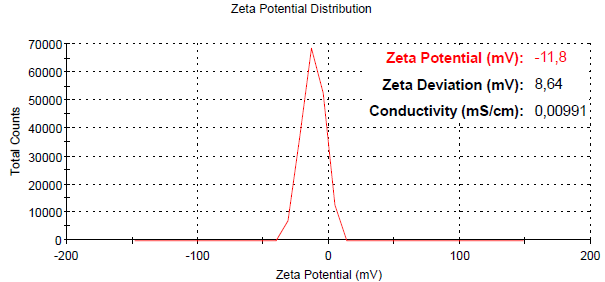
**

**1.50 ratio Curcumin/Gelatin (w/w)**

**PDI: 0.798, Kcps: 95, Z: 5267 nm, Avg: 663.82 nm**

**
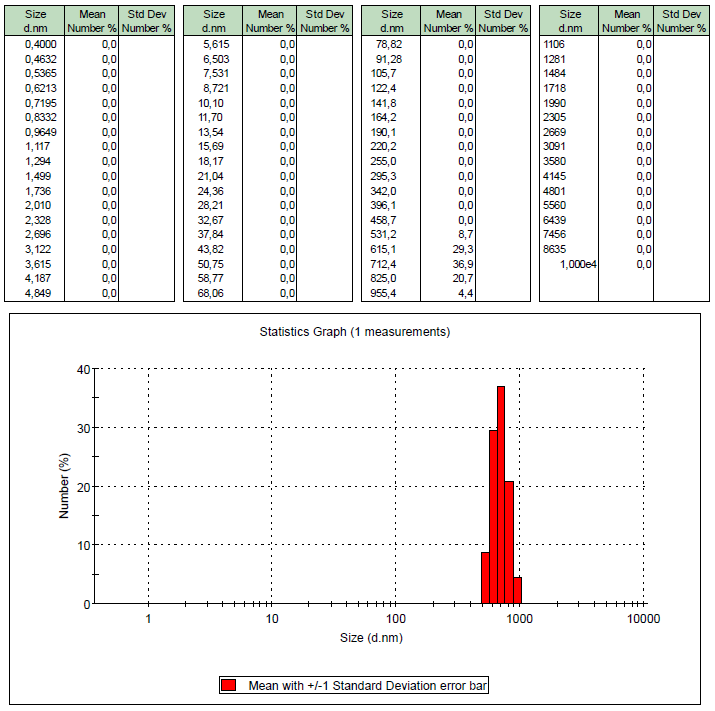
**

**
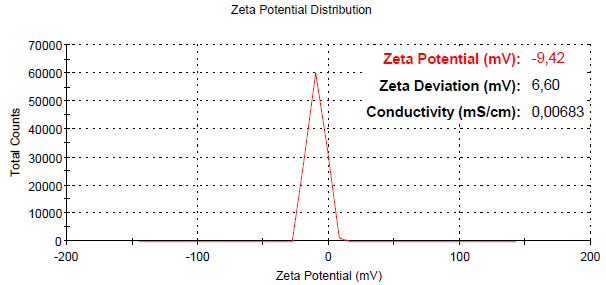
**
