# Supplementary material for: Synthesis and characterization of NIR-sensitive curcumin-gelatin nanoparticles for targeted drug delivery in 3D colon cancer
Source: Sci Rep. 2026 Mar 5;16:12167. doi: 10.1038/s41598-026-42199-3 (PMC13076676; doi:10.1038/s41598-026-42199-3)
Supplement: Supplementary file 2 — Supplementary Material 2 [file 41598_2026_42199_MOESM2_ESM.docx]

**Supplementary Material 2 for:**

**Synthesis and Characterization of NIR-Sensitive Curcumin-Gelatin Nanoparticles for Targeted Drug Delivery in 3D Colon Cancer**

Dilşad Özerkan^1*^, Ferdane Danışman-Kalındemirtaş^2*^, İshak Afşin Kariper^3^

^1*^ Kastamonu University, Faculty of Engineering and Architecture, Department of Genetic and Bioengineering, Kastamonu/TURKEY

^2*^Erzincan Binali Yıldırım University, Faculty of Medicine, Department of Physiology, Erzincan, TURKEY

^3^ Erciyes University, Education Faculty, Department of Science Education, Kayseri, TURKEY

**HPLC RESULTS**

**SONICATION**

**1 second: standard is 1000 ppm. Measured: 395 ppm %60.5 curcumin is adsorbed by gelatine.**

**
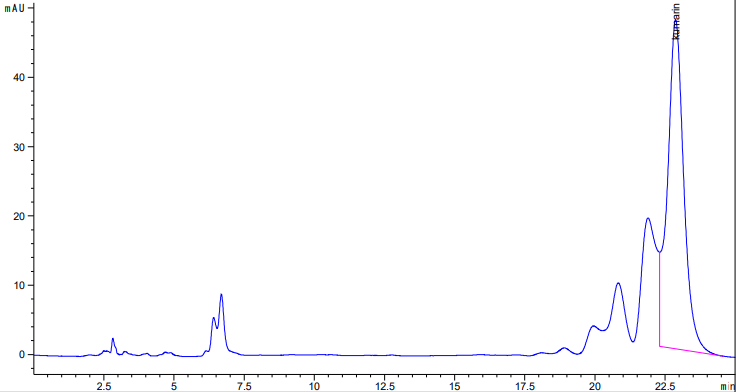
**

**5 seconds: standard is 1000 ppm. Measured: 388 ppm %61.2 curcumin is adsorbed by gelatine.**

**
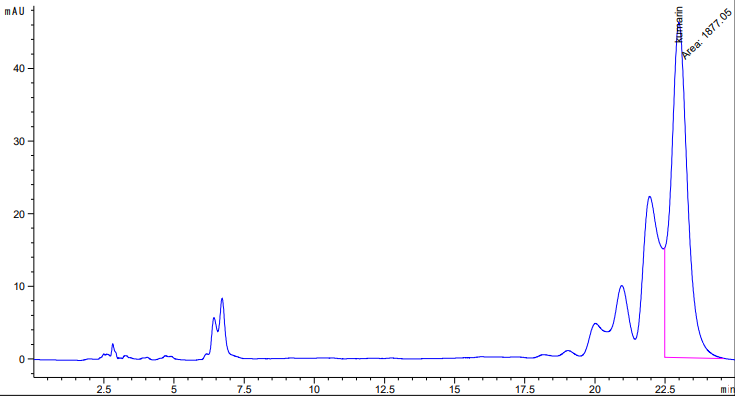
**

**10 seconds: standard is 1000 ppm. Measured: 384 ppm %61.6 curcumin is adsorbed by gelatine.**

**
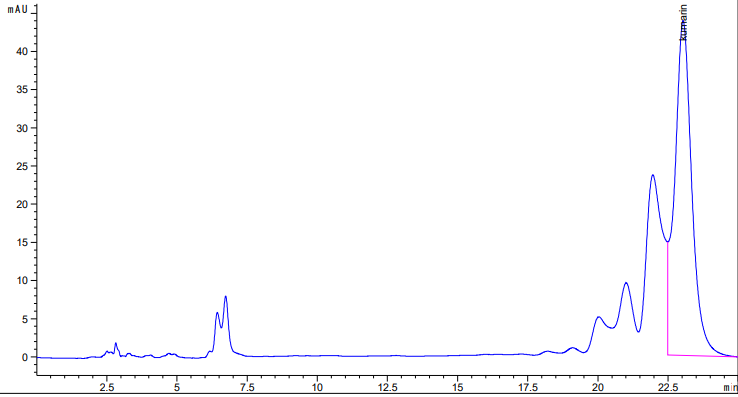
**

**20 seconds: standard is 1000 ppm. Measured: 376 ppm %62.4 curcumin is adsorbed by gelatine.**

**
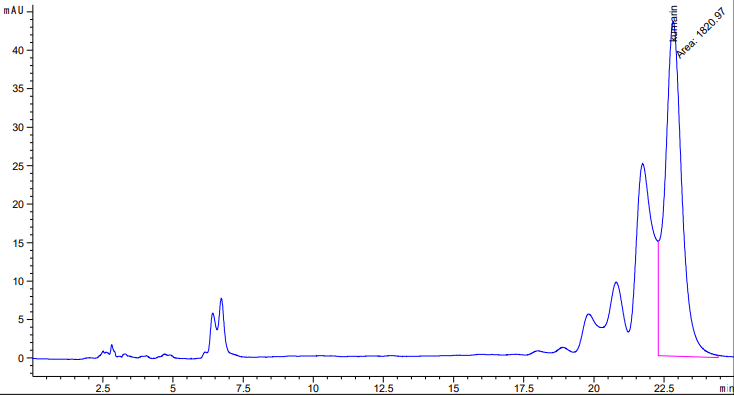
**

**30 seconds: standard is 1000 ppm. Measured: 360 ppm %64 curcumin is adsorbed by gelatine.**

**
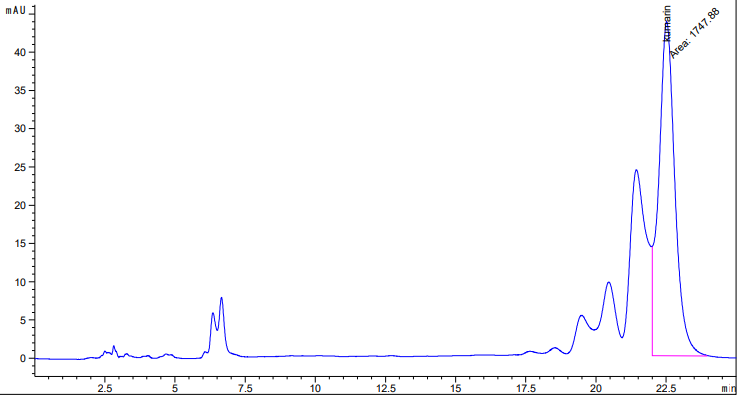
**

**60 seconds: standard is 1000 ppm. Measured: 369 ppm %63.1 curcumin is adsorbed by gelatine.**

**
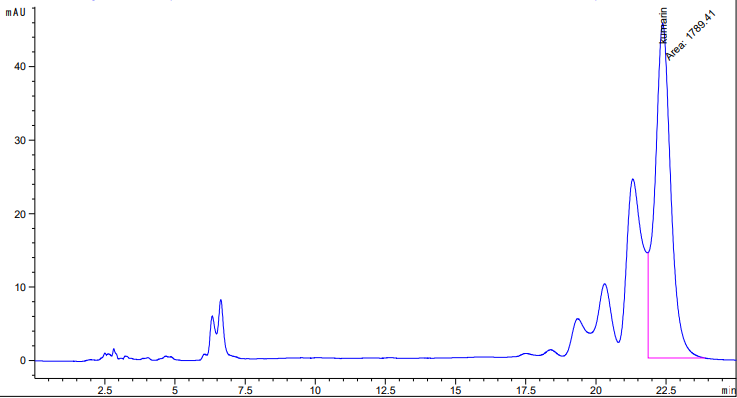
**

**120 seconds: standard is 1000 ppm. Measured: 378 ppm %62.2 curcumin is adsorbed by gelatine.**

**
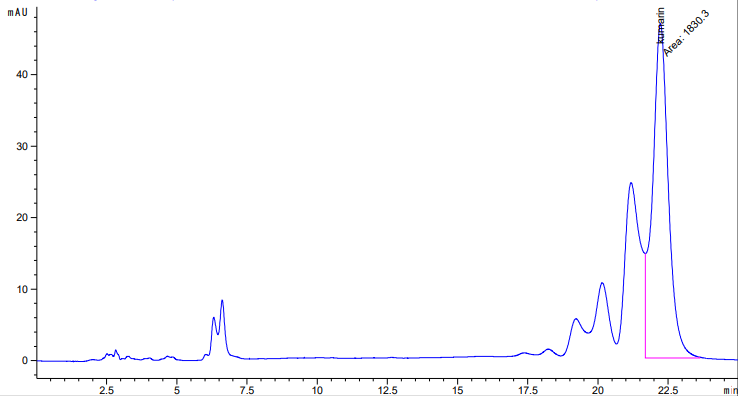
**

**180 seconds: standard is 1000 ppm. Measured: 396 ppm %60.4 curcumin is adsorbed by gelatine.**

**
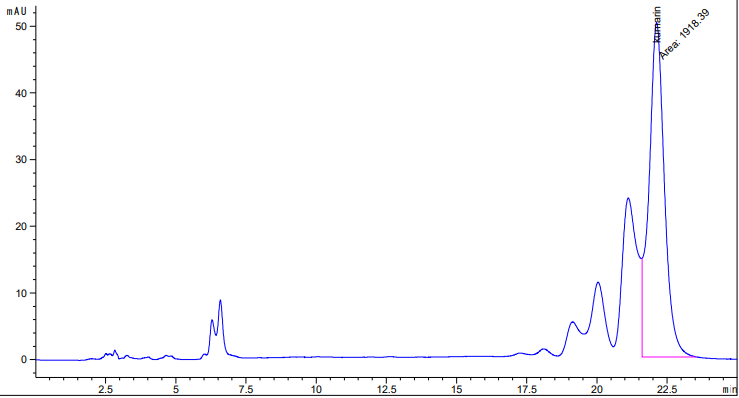
**

**CONCENTRATION**

**0.10 ratio Curcumin/Gelatin (w/w): standard is 100 ppm. Measured: 24 ppm %76 curcumin is adsorbed by gelatine.**

**
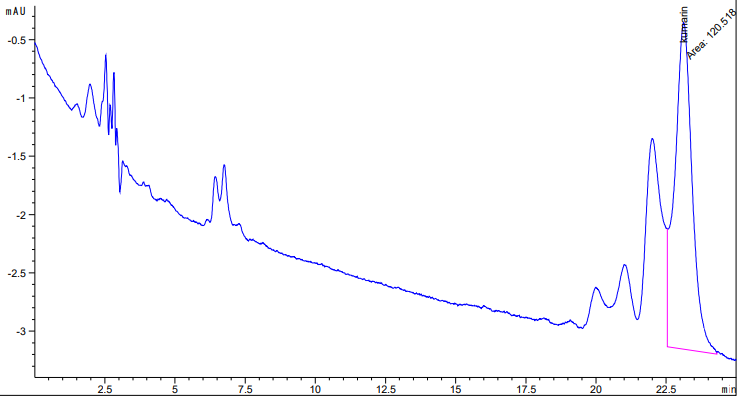
**

**0.25 ratio Curcumin/Gelatin (w/w): standard is 250 ppm. Measured: 109 ppm %56.4 curcumin is adsorbed by gelatine.**

**
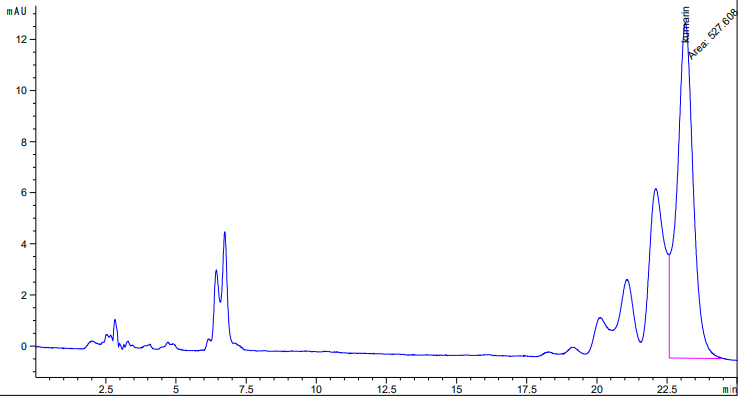
**

**0.50 ratio Curcumin/Gelatin (w/w): standard is 500 ppm. Measured: 255 ppm %49 curcumin is adsorbed by gelatine.**

**
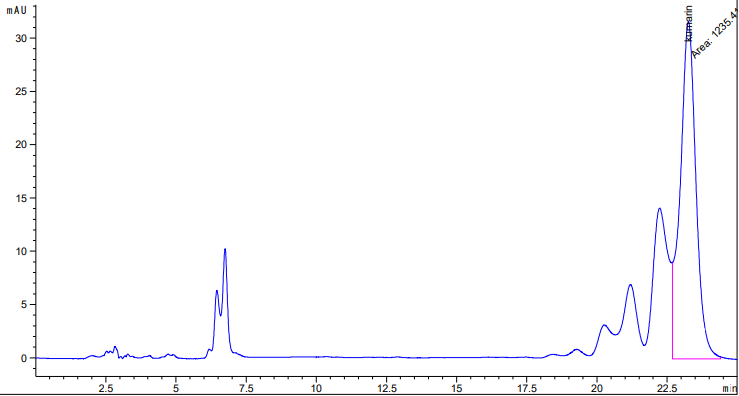
**

**0.75 ratio Curcumin/Gelatin (w/w): standard is 750 ppm. Measured: 399 ppm %53.2 curcumin is adsorbed by gelatine.**

**
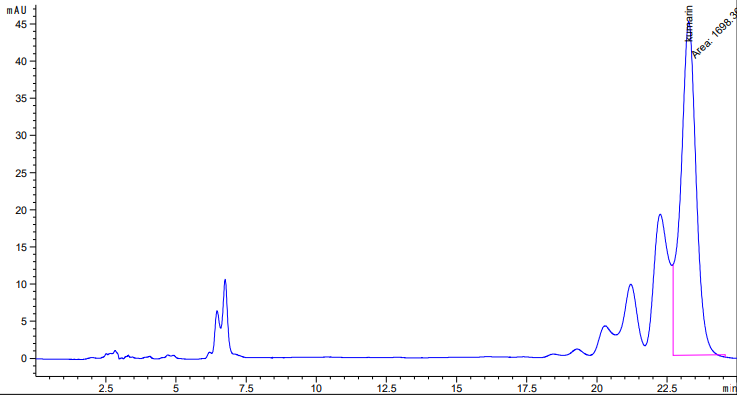
**

**1 ratio Curcumin/Gelatin (w/w): standard is 1000 ppm. Measured: 390 ppm %61 curcumin is adsorbed by gelatine.**

**
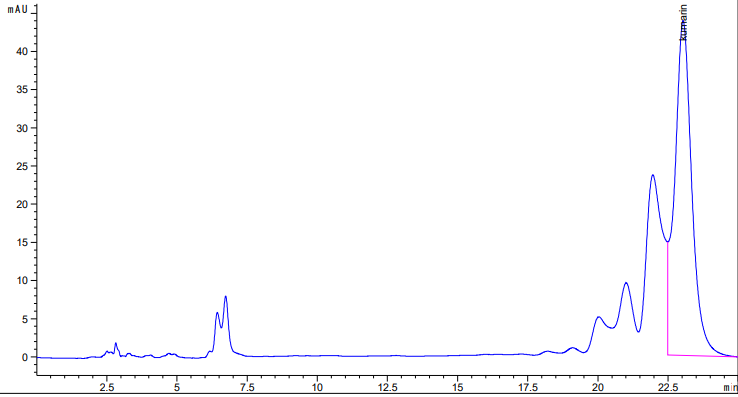
**

**1.25 ratio Curcumin/Gelatin (w/w): standard is 1250 ppm. Measured: 496 ppm %60.32 curcumin is adsorbed by gelatine.**

**
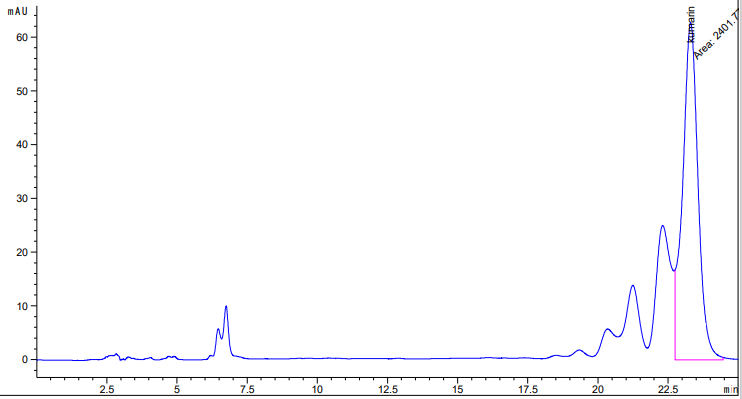
**

**1.50 ratio Curcumin/Gelatin (w/w): standard is 1500 ppm. Measured: 1071 ppm %28.6 curcumin is adsorbed by gelatine.**

**
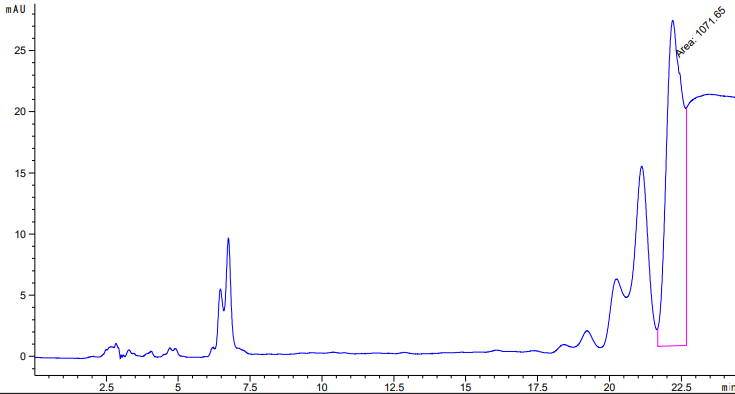
**
