# Supplementary material for: Synthesis and characterization of NIR-sensitive curcumin-gelatin nanoparticles for targeted drug delivery in 3D colon cancer
Source: Sci Rep. 2026 Mar 5;16:12167. doi: 10.1038/s41598-026-42199-3 (PMC13076676; doi:10.1038/s41598-026-42199-3)
Supplement: Supplementary file 3 — Supplementary Material 3 [file 41598_2026_42199_MOESM3_ESM.docx]

**Supplementary Material 3 for:**

**Synthesis and Characterization of NIR-Sensitive Curcumin-Gelatin Nanoparticles for Targeted Drug Delivery in 3D Colon Cancer**

Dilşad Özerkan^1*^, Ferdane Danışman-Kalındemirtaş^2*^, İshak Afşin Kariper^3^

^1*^ Kastamonu University, Faculty of Engineering and Architecture, Department of Genetic and Bioengineering, Kastamonu/TURKEY

^2*^Erzincan Binali Yıldırım University, Faculty of Medicine, Department of Physiology, Erzincan, TURKEY

^3^ Erciyes University, Education Faculty, Department of Science Education, Kayseri, TURKEY

**Activated by nanorobots through heat**

**DLS Results**

**37°C: average grain size: 54.23 nm, Z: 2209 nm, kcps: 224, PDI: 0.281**

**
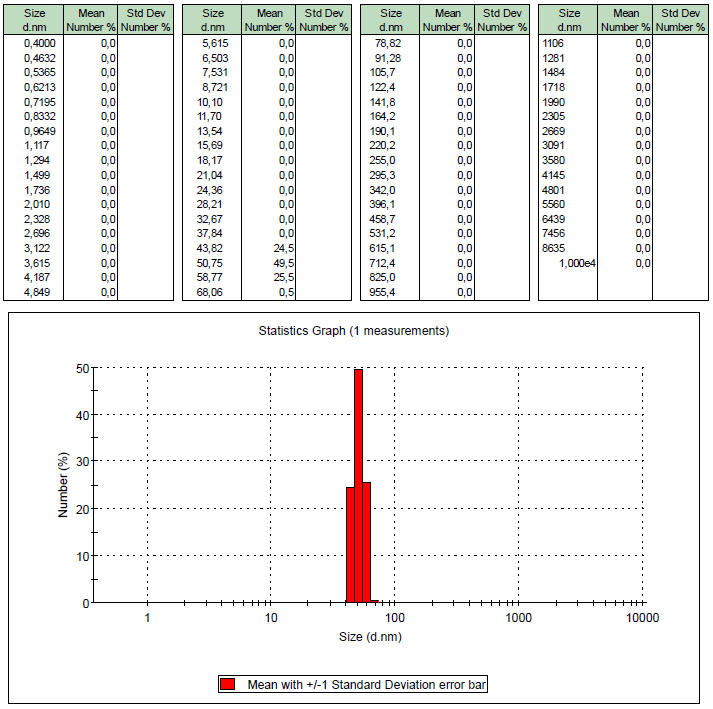
**

**
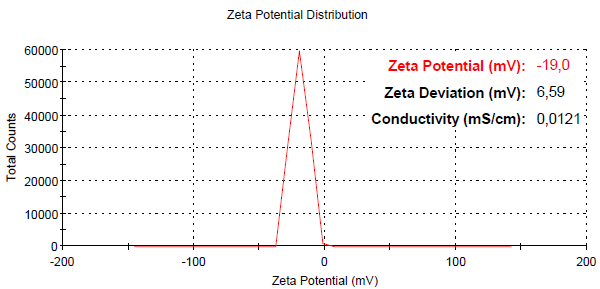
**

**38°C: average grain size: 79.23 nm, Z: 1212 nm, kcps: 428, PDI: 0.607**

**
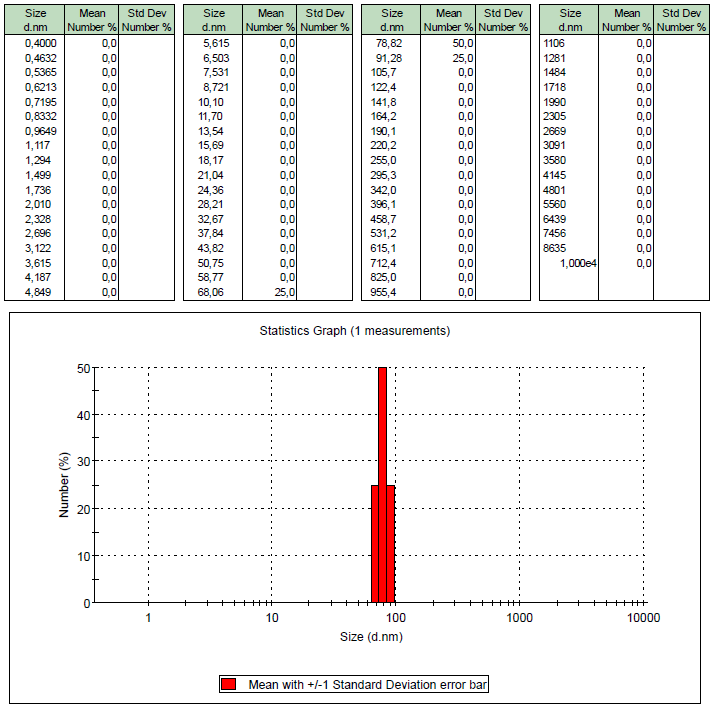
**

**
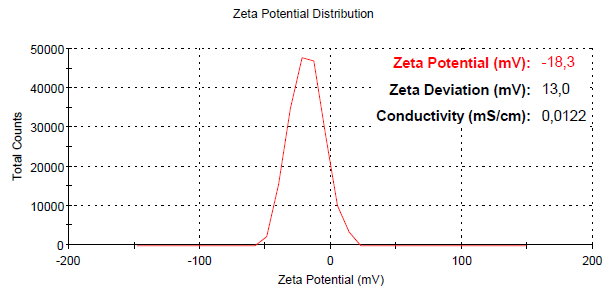
**

**39°C: average grain size: 158.71 nm, Z: 1640 nm, kcps: 60, PDI: 0.707**

**
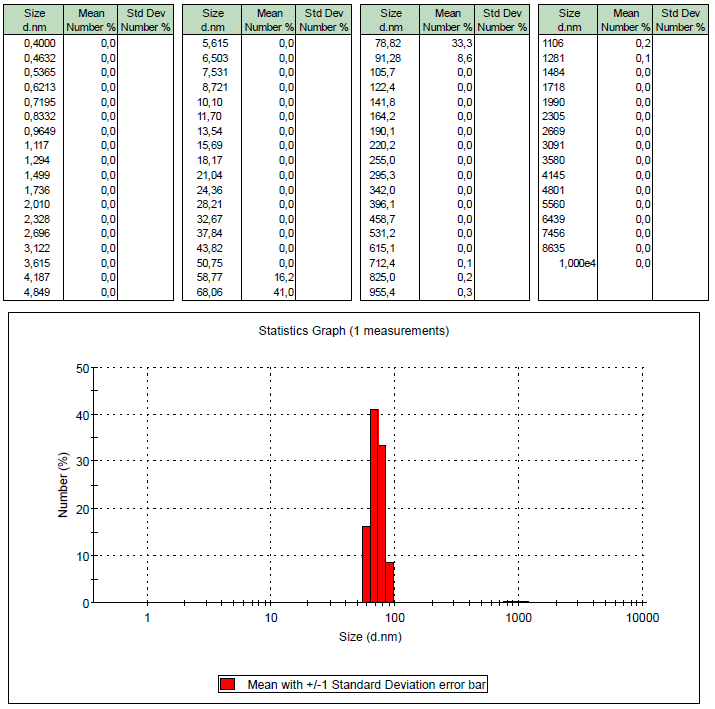
**

**
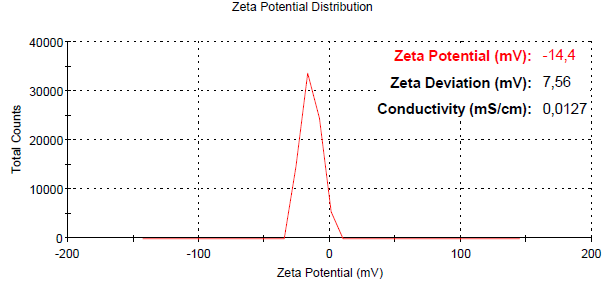
**

**40°C: average grain size: 157.64 nm, Z: 2065 nm, kcps: 78, PDI: 0.575**

**
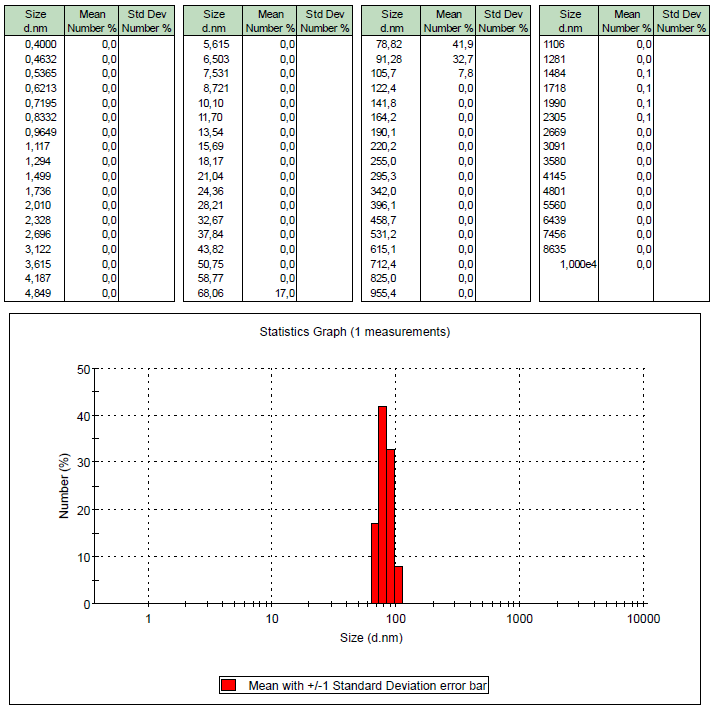
**

**
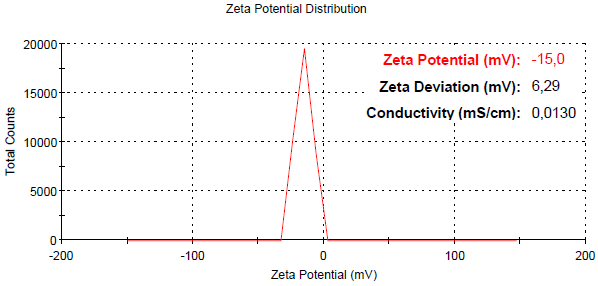
**

**41°C: average grain size: 395.96 nm, Z: 1514 nm, kcps: 78, PDI: 0.540**

**
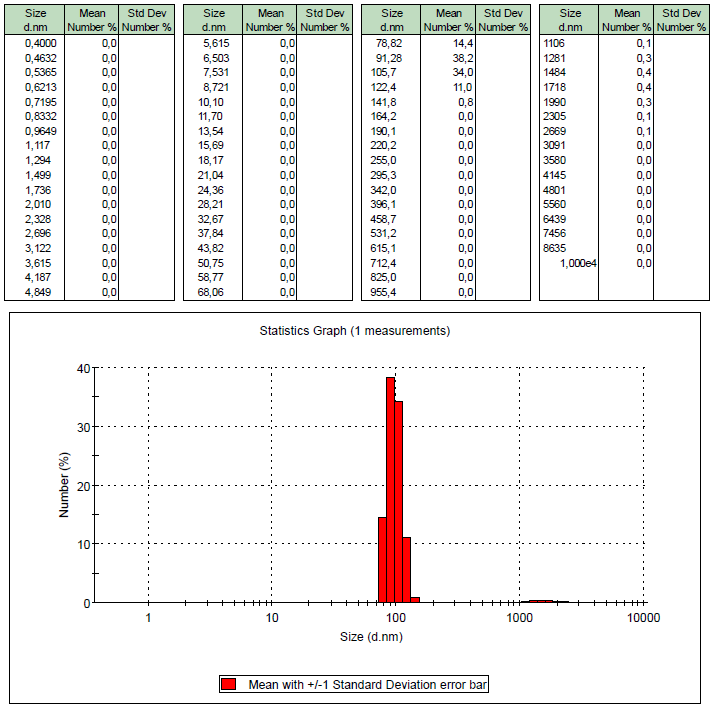
**

**
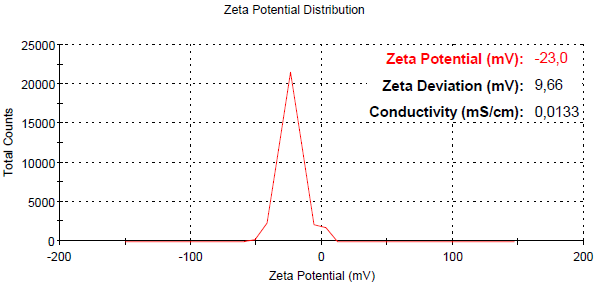
**

**42°C: average grain size: 206.27 nm, Z: 3658 nm, kcps: 393, PDI: 0.895**

**
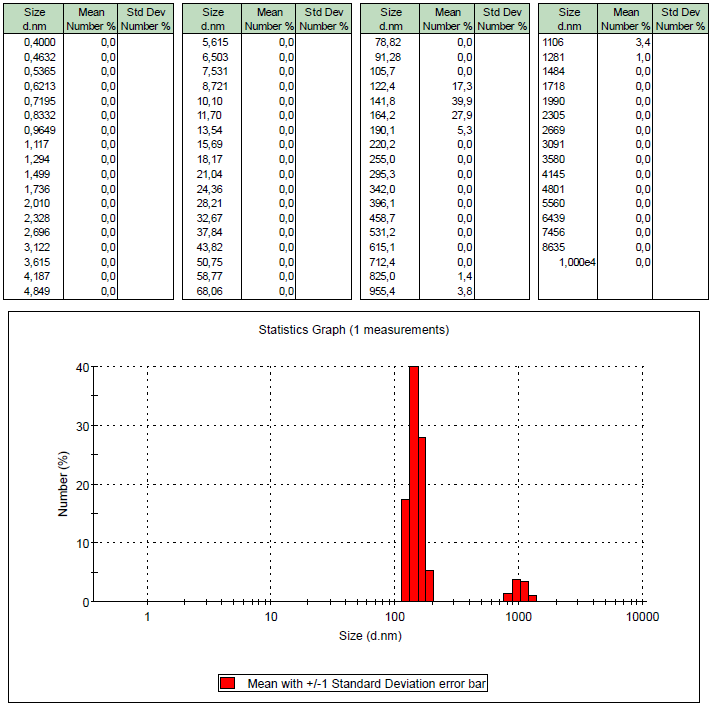
**

**
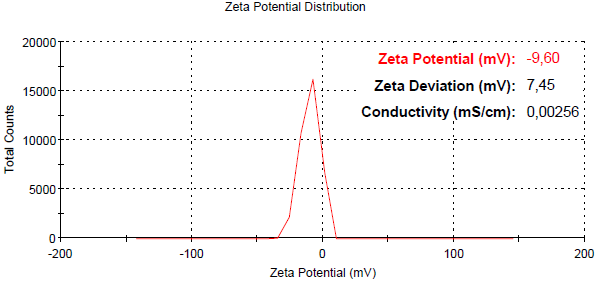
**

**HPLC Results:**

**37 °C: the standard is 500 ppm, as the carrier only adsorbs 64% of the drug. This means that 180 ppm curcumin is already present in the environment of the solution: 320 ppm curcumin is adsorbed by the gelatin. Measured: 335 ppm. 335 ppm – 180 ppm =155 ppm. 155 ppm/320 ppmx100= %48.43 Curcumin is adsorbed by gelatine.
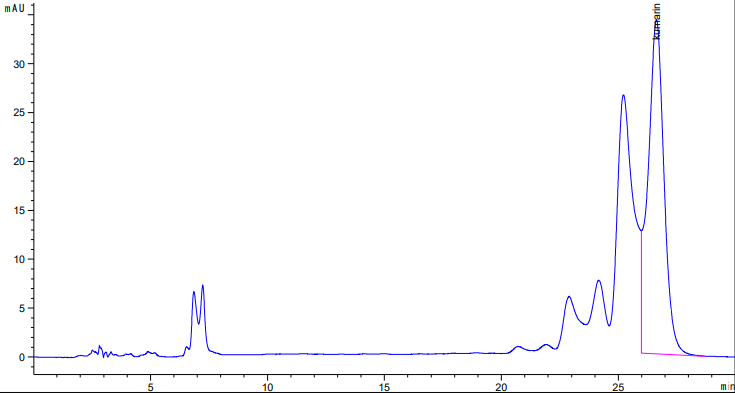
**

**38 °C: the standard is 500 ppm, as the carrier only adsorbs 64% of the drug. This means that 180 ppm curcumin is already present in the environment of the solution: 320 ppm curcumin is adsorbed by the gelatin. Measured: 338 ppm. 338 ppm-180 ppm=158 ppm. 158ppm/320ppmx100=%49.37 Curcumin is adsorbed by gelatine.**

**
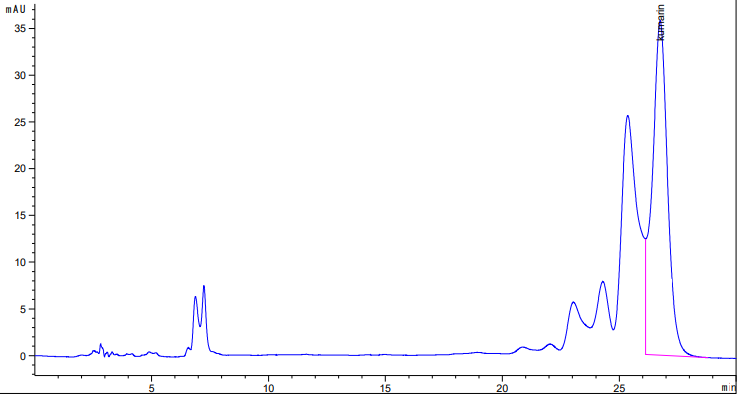
**

**39 °C: standard is 500 ppm. Measured: 329 ppm, as the carrier only adsorbs 64% of the active ingredient. This means that 180 ppm curcumin is already present in the environment of the solution: 320 ppm curcumin is adsorbed by the gelatine. 329 ppm-180 ppm= 149 ppm. 149 ppm/320 ppmx100= %46.56 Curcumin is adsorbed by gelatine.**

**
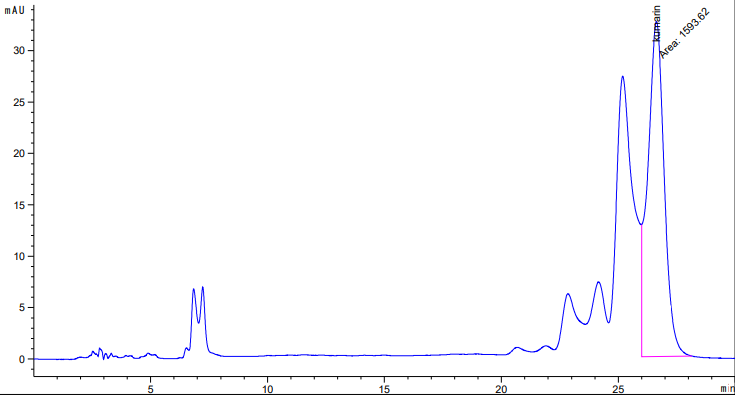
**

**40 °C: standard curcumin is 500 ppm. Measured: 325 ppm curcumin, as the carrier only adsorbs 64% of the active ingredient. This means that 180 ppm curcumin is already in the solution environment: 320 ppm curcumin is adsorbed by the gelatine. 325 ppm – 180 ppm = 145 ppm. 145 ppm/320 ppmx100 = %45.31 Curcumin is adsorbed by gelatine.**

**
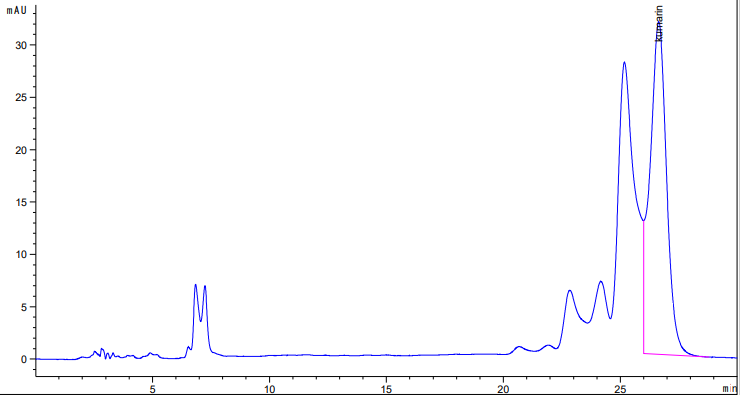
**

**41 °C:** the standard curve is 500 ppm. Measured: 315 ppm curcumin in the solution. However, since the carrier only adsorbs 64% of the active ingredient. This means that 180 ppm curcumin is already in the environment of the solution: 320 ppm curcumin is adsorbed by the gelatine. 315 ppm – 180 ppm = 135 ppm curcumin is adsorbed by the gelatine. 135 ppm / 320 ppm x100 = %42.18 Curcumin is adsorbed by gelatine.

**
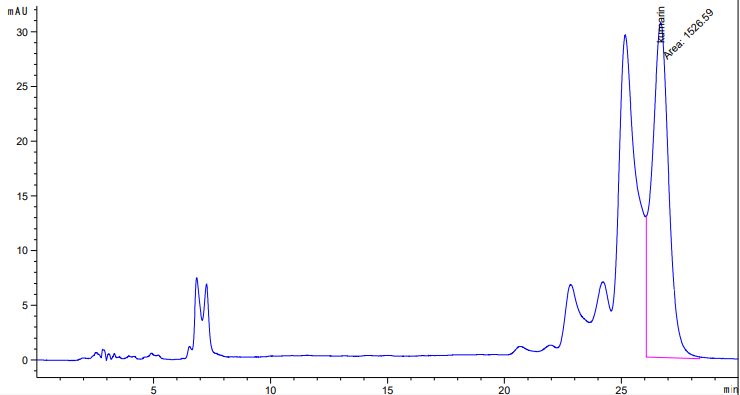
**

**42 °C: standard is 500 ppm. Measured: 334 ppm curcumin in the solution. However, since the carrier adsorbs only 64% of the drug. This means that 180 ppm curcumin is already present in the environment of the solution: 320 ppm curcumin is adsorbed by the gelatine. 334 ppm – 180 ppm = 154 ppm curcumin is adsorbed by the gelatine. 154 ppm / 320 ppm x100 = %48.12 Curcumin is adsorbed by gelatine.**

**
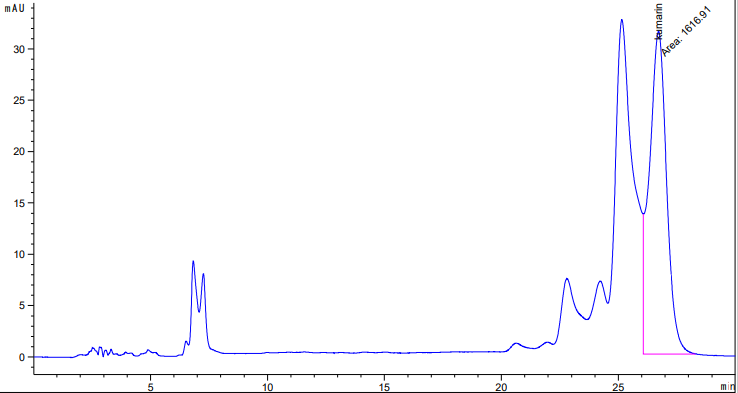
**
