# Supplementary material for: Synthesis and characterization of NIR-sensitive curcumin-gelatin nanoparticles for targeted drug delivery in 3D colon cancer
Source: Sci Rep. 2026 Mar 5;16:12167. doi: 10.1038/s41598-026-42199-3 (PMC13076676; doi:10.1038/s41598-026-42199-3)

**Supplementary Material 5 for:**

**Synthesis and Characterization of NIR-Sensitive Curcumin-Gelatin Nanoparticles for Targeted Drug Delivery in 3D Colon Cancer**

Dilşad Özerkan^1*^, Ferdane Danışman-Kalındemirtaş^2*^, İshak Afşin Kariper^3^

^1*^ Kastamonu University, Faculty of Engineering and Architecture, Department of Genetic and Bioengineering, Kastamonu/TURKEY

^2*^Erzincan Binali Yıldırım University, Faculty of Medicine, Department of Physiology, Erzincan, TURKEY

^3^ Erciyes University, Education Faculty, Department of Science Education, Kayseri, TURKEY

**HPLC Results**

**Activated Nanorobot**

**30 minutes: Standard curve is 500 ppm. Measured: 340 ppm curcumin in the solution. However, since the carrier only adsorbs 64% of the active ingredient. This means that 180 ppm curcumin is already in the environment of the solution: 320 ppm curcumin is adsorbed by the gelatine. 340 ppm – 180 ppm = 160 ppm curcumin is released from the gelatine. 160 ppm / 320 ppm x100 = %50 Curcumin is released by gelatine. The remaining amount of active ingredient: 250 ppm, %50**

**
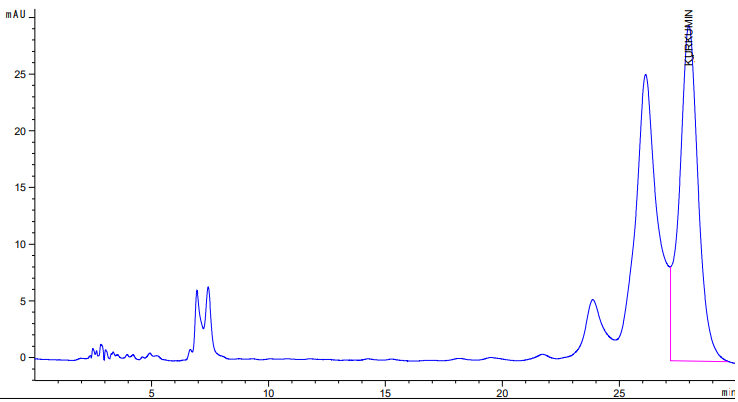
**

**1 hour: Standard curve is 500 ppm. Measured: 318 ppm curcumin in the solution. However, since the carrier only adsorbs 64% of the active ingredient. This means that 180 ppm curcumin is already in the environment of the solution: 320 ppm curcumin is adsorbed by the gelatine. 320 ppm – 180 ppm = 140 ppm curcumin is released from the gelatine. 140 ppm / 320 ppm x100 = %43.75 Curcumin is released by gelatine. The remaining amount of active ingredient: 140,625 ppm, %21.875**

**
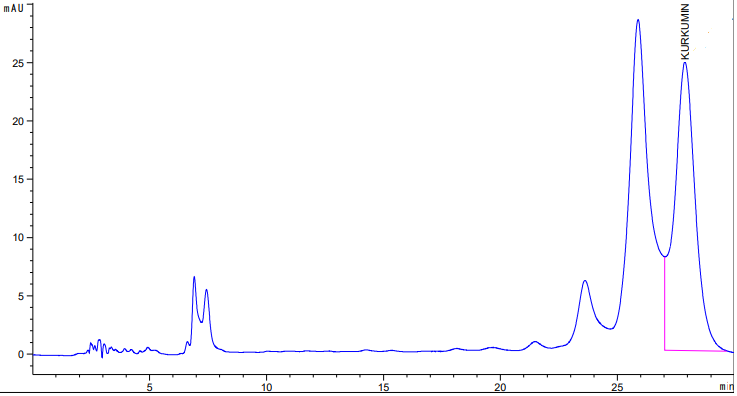
**

**2 hours: Standard curcumn is 500 ppm. Measured: 280 ppm curcumin in the solution. However, since the carrier only adsorbs 64% of the active ingredient. This means that 180 ppm curcumin is already present in the environment of the solution: 320 ppm curcumin is adsorbed by the gelatin. 280 ppm – 180 ppm = 100 ppm curcumin is released from the gelatine. 100 ppm / 320 ppm x100 = %31.25 Curcumin is released by gelatine. The remaining amount of active ingredient: 96.67 ppm, %19.334**


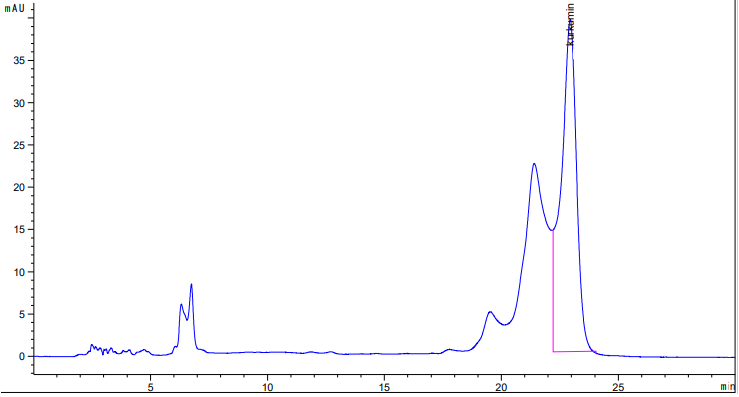


**4 hours: Standard curcumn is 500 ppm. Measured: 294 ppm curcumin in the solution. However, since the carrier only adsorbs 64% of the active ingredient. This means that 180 ppm curcumin is already present in the environment of the solution: 320 ppm curcumin is adsorbed by the gelatin. 294 ppm – 180 ppm = 114 ppm curcumin is released from the gelatine. 114 ppm / 320 ppm x100 = %35.63 Curcumin is released by gelatine. The remaining amount of active ingredient: 62.22 ppm, %12.44**


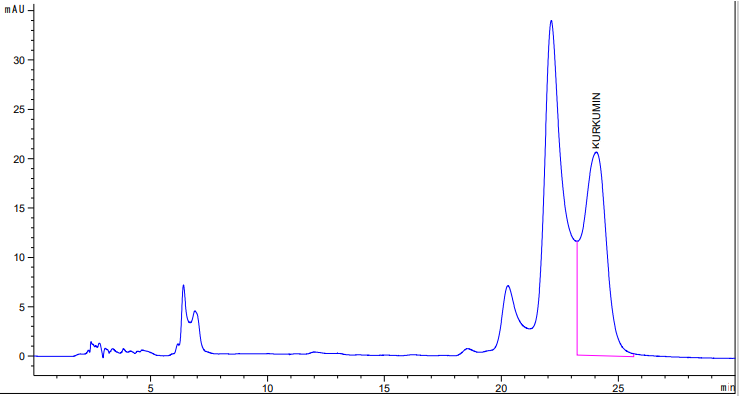


**6 hours: Standard curcumin is 500 ppm. Measured: 249 ppm curcumin in the solution. However, since the carrier only absorbs 64% of the active ingredient. This means that 180 ppm curcumin is already in the environment of the solution: 320 ppm curcumin is adsorbed by the gelatine. 249 ppm – 180 ppm = 69 ppm curcumin is released from the gelatine. 69 ppm / 320 ppm x100 = %21.56 Curcumin is released by gelatine. The remaining amount of active ingredient: 48.80 ppm, %9.76**


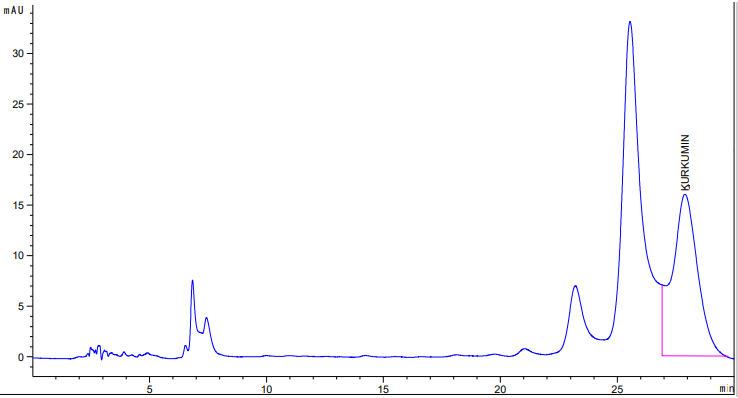


**24 hours: standard curcumn is 500 ppm. Measured: 251 ppm curcumin in the solution. However since the carrier only adsorbs 64% of the drug. That is, since 180 ppm curcumin is already in the solution environment: 320 ppm curcumin is adsorbed by gelatin. 251 ppm – 180 ppm = 71 ppm curcumin is released by gelatin. 71 ppm / 320 ppm x100 = %22.19 curcumin is released by gelatin. The remaining amount of drug: 37.97 ppm, %7.59**
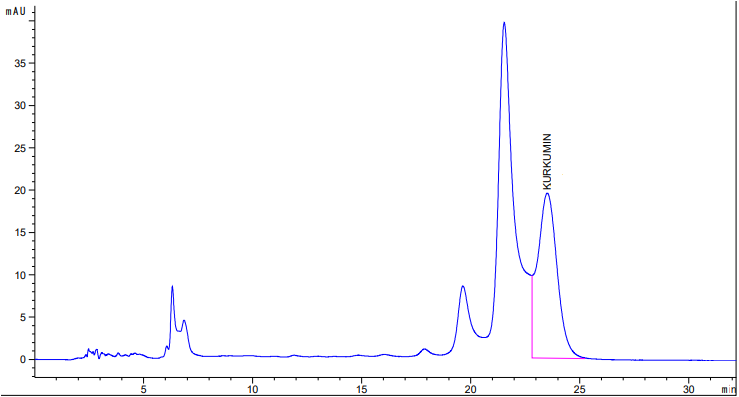


**Un-Activated Nanorobot**

**30 minutes: Standard curcumin is 500 ppm. Measured: 301 ppm curcumin in the solution. However, since the carrier only adsorbs 64% of the active ingredient. This means that 180 ppm curcumin is already in the environment of the solution: 320 ppm curcumin is adsorbed by the gelatine. 301 ppm – 180 ppm = 121 ppm curcumin is released from the gelatine. 121 ppm / 320 ppm x100 = %37.81 Curcumin is released by gelatine. The remaining amount of active ingredient: 311 ppm, %62.20**


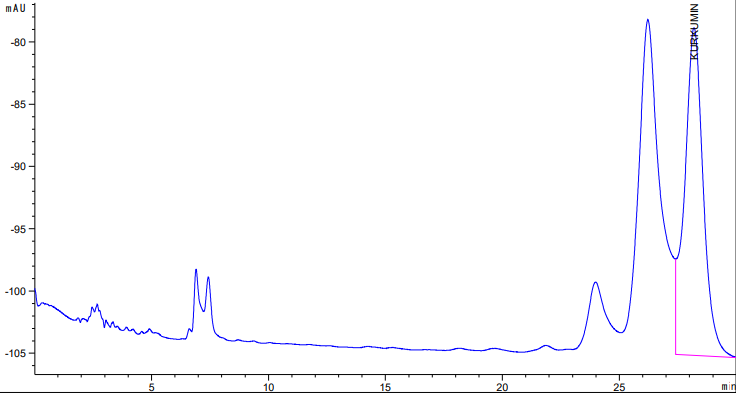


**1 hour: Standard curve is 500 ppm. Measured: 316 ppm curcumin in the solution. However, since the carrier only adsorbs 64% of the active ingredient. This means that 180 ppm curcumin is already in the environment of the solution: 320 ppm curcumin is adsorbed by the gelatine. 316 ppm – 180 ppm = 136 ppm curcumin is released from the gelatine. 136 ppm / 320 ppm x100 = %42.50 Curcumin is released by gelatine. The remaining amount of active ingredient: 178.82 ppm, %32.76**

**
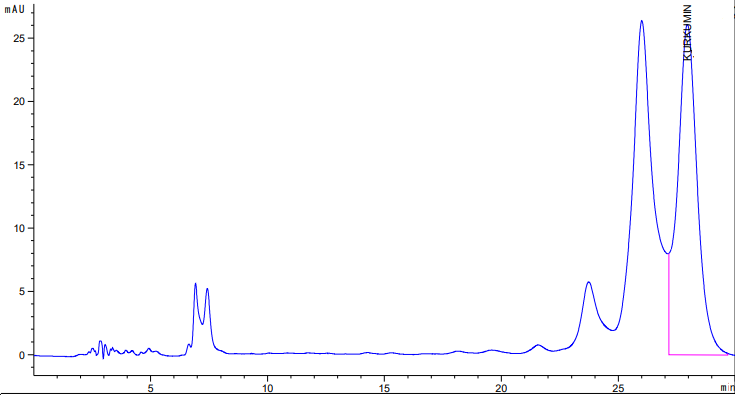
**

**2 hours: Standard curcumn is 500 ppm. Measured: 245 ppm curcumin in the solution. However, since the carrier only adsorbs 64% of the active ingredient. This means that 180 ppm curcumin is already in the environment of the solution: 320 ppm curcumin is adsorbed by the gelatine. 245 ppm – 180 ppm = 65 ppm curcumin is released from the gelatine. 65 ppm / 320 ppm x100 = %20.31 Curcumin is released by gelatine. The remaining amount of active ingredient: 142.50 ppm, %28.50**

**
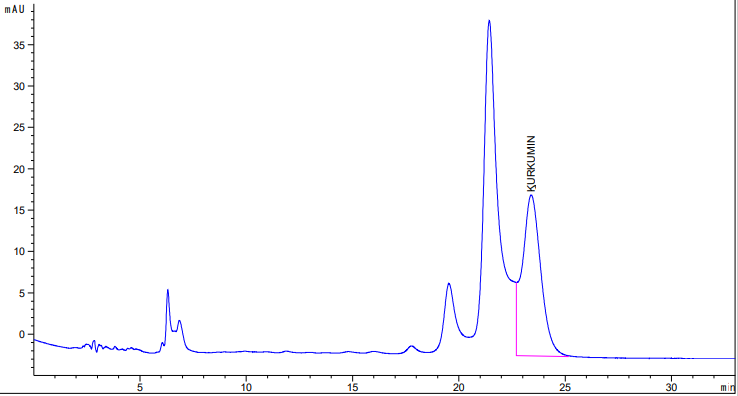
**

**4 hours: Standard curcum is 500 ppm. Measured: 259 ppm curcumin in the solution. However, since the carrier only adsorbs 64% of the active ingredient. This means that 180 ppm curcumin is already in the environment of the solution: 320 ppm curcumin is adsorbed by the gelatine. 259 ppm – 180 ppm = 79 ppm curcumin is released from the gelatine. 79 ppm / 320 ppm x100 = %24.68 Curcumin is released by gelatine. The remaining amount of active ingredient: 107.33 ppm, %21.47**


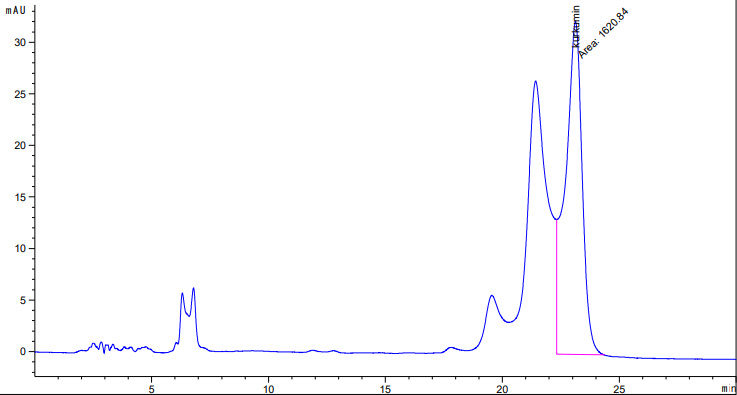


**6 hours: Standard curcumn is 500 ppm. Measured: 221 ppm curcumin in the solution. However, since the carrier only adsorbs 64% of the active ingredient. This means that 180 ppm curcumin is already in the environment of the solution: 320 ppm curcumin is adsorbed by the gelatine. 221 ppm – 180 ppm = 31 ppm curcumin is released from the gelatine. 31 ppm / 320 ppm x100 = %9.68 Curcumin is released by gelatine. The remaining amount of active ingredient: 96.94 ppm, %19.38**


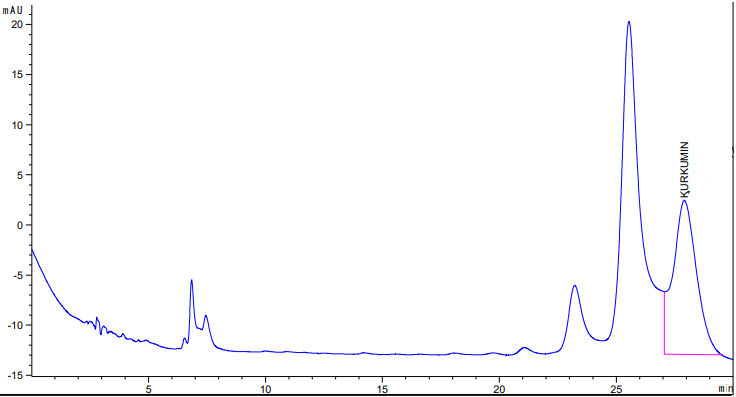


**24 hours: Standard curcum is 500 ppm. Measured: 199 ppm curcumin in the solution. However, since the carrier only adsorbs 64% of the active ingredient. This means that 180 ppm curcumin is already in the environment of the solution: 320 ppm curcumin is adsorbed by the gelatine. 221 ppm – 180 ppm = 19 ppm curcumin is released from the gelatine. 19 ppm / 320 ppm x100 = %5.93 Curcumin is released by gelatine. The remaining amount of active ingredient: 91.20 ppm, %18.24**


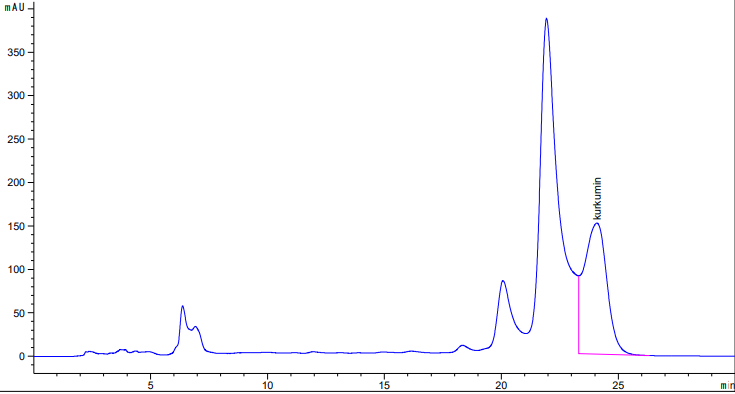

Supplement: Supplementary file 4 — Supplementary Material 4 [file 41598_2026_42199_MOESM4_ESM.docx]
