# Supplementary material for: Synthesis and characterization of NIR-sensitive curcumin-gelatin nanoparticles for targeted drug delivery in 3D colon cancer
Source: Sci Rep. 2026 Mar 5;16:12167. doi: 10.1038/s41598-026-42199-3 (PMC13076676; doi:10.1038/s41598-026-42199-3)

**Supplementary Material 6 for:**

**Synthesis and Characterization of NIR-Sensitive Curcumin-Gelatin Nanoparticles for Targeted Drug Delivery in 3D Colon Cancer**

Dilşad Özerkan^1*^, Ferdane Danışman-Kalındemirtaş^2*^, İshak Afşin Kariper^3^

^1*^ Kastamonu University, Faculty of Engineering and Architecture, Department of Genetic and Bioengineering, Kastamonu/TURKEY

^2*^Erzincan Binali Yıldırım University, Faculty of Medicine, Department of Physiology, Erzincan, TURKEY

^3^ Erciyes University, Education Faculty, Department of Science Education, Kayseri, TURKEY

**Drug release from nanorobots with membrane**

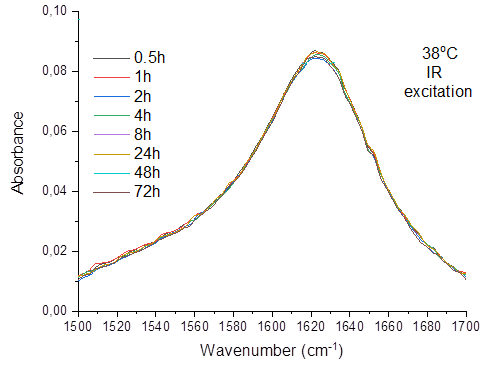

Supplement: Supplementary file 6 — Supplementary Material 6 [file 41598_2026_42199_MOESM6_ESM.docx]
