# Supplementary material for: Synthesis and characterization of NIR-sensitive curcumin-gelatin nanoparticles for targeted drug delivery in 3D colon cancer
Source: Sci Rep. 2026 Mar 5;16:12167. doi: 10.1038/s41598-026-42199-3 (PMC13076676; doi:10.1038/s41598-026-42199-3)
Supplement: Supplementary file 7 — Supplementary Material 7 [file 41598_2026_42199_MOESM7_ESM.docx]

**Supplementary Material 7 for:**

**Synthesis and Characterization of NIR-Sensitive Curcumin-Gelatin Nanoparticles for Targeted Drug Delivery in 3D Colon Cancer**

Dilşad Özerkan^1*^, Ferdane Danışman-Kalındemirtaş^2*^, İshak Afşin Kariper^3^

^1*^ Kastamonu University, Faculty of Engineering and Architecture, Department of Genetic and Bioengineering, Kastamonu/TURKEY

^2*^Erzincan Binali Yıldırım University, Faculty of Medicine, Department of Physiology, Erzincan, TURKEY

^3^ Erciyes University, Education Faculty, Department of Science Education, Kayseri, TURKEY


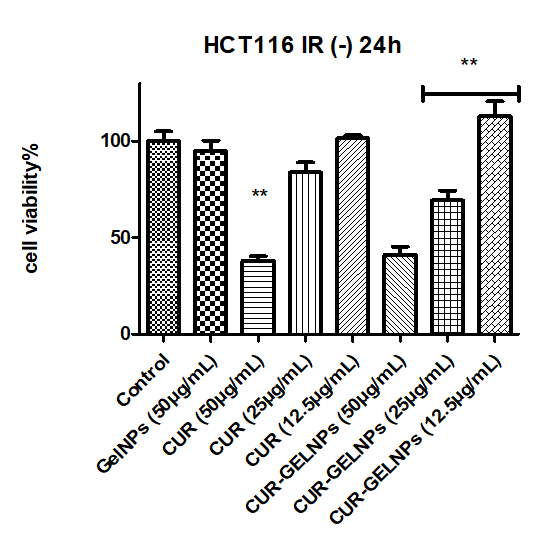

**Figure S1.** The effect of different doses of Cur-GelNPs on the viability of IR-treated HCT116 colon cancer cells is shown. (Data are presented as mean ± SEM, n = 3, ** 0.0048 p<0.005; * 0.0298 p<0.05)

**Figure S2.** The effect of different doses of Cur-GelNPs on the viability of IR-treated HT29 colon cancer cells is shown. (Data are presented as mean ± SEM, n = 3, ** 0.0048 p<0.005; * 0.0298 p<0.05)

**Figure S3.** The effect of different doses of Cur-GelNPs on the viability of IR-treated HUVEC healthy endothelial cells is shown. (Data are presented as mean ± SEM, n = 3, ** 0.0048 p<0.005; * 0.0298 p<0.05)
